# Surveillance for Violent Deaths — National Violent Death Reporting System, 48 States, the District of Columbia, and Puerto Rico, 2020

**DOI:** 10.15585/mmwr.ss7205a1

**Published:** 2023-05-26

**Authors:** Grace S. Liu, Brenda L. Nguyen, Bridget H. Lyons, Kameron J. Sheats, Rebecca F. Wilson, Carter J. Betz, Katherine A. Fowler

**Affiliations:** 1 Division of Violence Prevention, National Center for Injury Prevention and Control, CDC

## Abstract

**Problem/Condition:**

In 2020, approximately 71,000 persons died of violence-related injuries in the United States. This report summarizes data from CDC’s National Violent Death Reporting System (NVDRS) on violent deaths that occurred in 48 states, the District of Columbia, and Puerto Rico in 2020. Results are reported by sex, age group, race and ethnicity, method of injury, type of location where the injury occurred, circumstances of injury, and other selected characteristics.

**Period Covered:**

2020.

**Description of System:**

NVDRS collects data regarding violent deaths obtained from death certificates, coroner and medical examiner records, and law enforcement reports. This report includes data collected for violent deaths that occurred in 2020. Data were collected from 48 states (all states with exception of Florida and Hawaii), the District of Columbia, and Puerto Rico. Forty-six states had statewide data, two additional states had data from counties representing a subset of their population (35 California counties, representing 71% of its population, and four Texas counties, representing 39% of its population), and the District of Columbia and Puerto Rico had jurisdiction-wide data. NVDRS collates information for each violent death and links deaths that are related (e.g., multiple homicides, homicide followed by suicide, or multiple suicides) into a single incident.

**Results:**

For 2020, NVDRS collected information on 64,388 fatal incidents involving 66,017 deaths that occurred in 48 states (46 states collecting statewide data, 35 California counties, and four Texas counties), and the District of Columbia. In addition, information was collected for 729 fatal incidents involving 790 deaths in Puerto Rico. Data for Puerto Rico were analyzed separately. Of the 66,017 deaths, the majority (58.4%) were suicides, followed by homicides (31.3%), deaths of undetermined intent (8.2%), legal intervention deaths (1.3%) (i.e., deaths caused by law enforcement and other persons with legal authority to use deadly force acting in the line of duty, excluding legal executions), and unintentional firearm deaths (<1.0%). The term “legal intervention” is a classification incorporated into the *International Classification of Diseases, Tenth Revision*, and does not denote the lawfulness or legality of the circumstances surrounding a death caused by law enforcement.

Demographic patterns and circumstances varied by manner of death. The suicide rate was higher for males than for females. Across all age groups, the suicide rate was highest among adults aged ≥85 years. In addition, non-Hispanic American Indian or Alaska Native (AI/AN) persons had the highest suicide rates among all racial and ethnic groups. Among both males and females, the most common method of injury for suicide was a firearm. Among all suicide victims, when circumstances were known, suicide was most often preceded by a mental health, intimate partner, or physical health problem or by a recent or impending crisis during the previous or upcoming 2 weeks. The homicide rate was higher for males than for females. Among all homicide victims, the homicide rate was highest among persons aged 20–24 years compared with other age groups. Non-Hispanic Black (Black) males experienced the highest homicide rate of any racial or ethnic group. Among all homicide victims, the most common method of injury was a firearm. When the relationship between a homicide victim and a suspect was known, the suspect was most frequently an acquaintance or friend for male victims and a current or former intimate partner for female victims. Homicide most often was precipitated by an argument or conflict, occurred in conjunction with another crime, or, for female victims, was related to intimate partner violence. Nearly all victims of legal intervention deaths were male, and the legal intervention death rate was highest among men aged 35–44 years. The legal intervention death rate was highest among AI/AN males, followed by Black males. A firearm was used in the majority of legal intervention deaths. When a specific type of crime was known to have precipitated a legal intervention death, the type of crime was most frequently assault or homicide. When circumstances were known, the three most frequent circumstances reported for legal intervention deaths were as follows: the victim’s death was precipitated by another crime, the victim used a weapon in the incident, and the victim had a substance use problem (other than alcohol use).

Other causes of death included unintentional firearm deaths and deaths of undetermined intent. Unintentional firearm deaths were most frequently experienced by males, non-Hispanic White (White) persons, and persons aged 15–24 years. These deaths most frequently occurred while the shooter was playing with a firearm and were precipitated by a person unintentionally pulling the trigger. The rate of deaths of undetermined intent was highest among males, particularly among AI/AN and Black males, and among adults aged 30–54 years. Poisoning was the most common method of injury in deaths of undetermined intent, and opioids were detected in nearly 80% of decedents tested for those substances.

**Interpretation:**

This report provides a detailed summary of data from NVDRS on violent deaths that occurred in 2020. The suicide rate was highest among AI/AN and White males, whereas the homicide rate was highest among Black male victims. Intimate partner violence precipitated a large proportion of homicides for females. Mental health problems, intimate partner problems, interpersonal conflicts, and acute life stressors were primary circumstances for multiple types of violent death.

**Public Health Action:**

Violence is preventable, and states and communities can use data to guide public health action. NVDRS data are used to monitor the occurrence of violence-related fatal injuries and assist public health authorities in developing, implementing, and evaluating programs, policies, and practices to reduce and prevent violent deaths. For example, the Colorado Violent Death Reporting System (VDRS), Kentucky VDRS, and Oregon VDRS have used their VDRS data to guide suicide prevention efforts and generate reports highlighting where additional focus is needed. In Colorado, VDRS data were used to examine the increased risk for suicide among first and last responders in the state. Kentucky VDRS used local data to highlight how psychological and social effects of the COVID-19 pandemic might increase risk for suicide, particularly among vulnerable populations. Oregon VDRS used their data to develop a publicly available data dashboard displaying firearm mortality trends and rates in support of the state’s firearm safety campaign. Similarly, states participating in NVDRS have used their VDRS data to examine homicide in their state. Illinois VDRS, for example, found that state budget cuts were associated with notable increases in homicides among youths in Chicago. With an increase of participating states and jurisdictions, this report marks progress toward providing nationally representative data.

## Introduction

According to National Vital Statistics System mortality data obtained from CDC’s Web-based Injury Statistics Query and Reporting System (WISQARS),* violence-related injuries led to 71,335 deaths in the United States in 2020 (*1*). Suicide was the 12th leading cause of death overall in the United States and disproportionately affected young and middle-aged populations. By age group, suicide was among the three leading causes of death for persons aged 10–34 years and was the fourth leading cause of death among adults aged 35–44 years. Non-Hispanic American Indian or Alaska Native (AI/AN) and non-Hispanic White (White) males had the highest rates of suicide compared with all other racial and ethnic groups and females.

In 2020, homicide was the 16th leading cause of death overall in the United States but disproportionately affected young persons and non-Hispanic Black (Black) males (*1*). Homicide was among the four leading causes of death for children aged 1–14 years and was the second leading cause of death for persons aged 15–24 years and the third leading cause of death for persons aged 25–34 years. Homicide was the leading cause of death for Black males aged 15–24 years and the second leading cause of death for Black males aged 1–14 years.

Public health authorities require accurate, timely, and complete surveillance data to better understand and ultimately prevent the occurrence of violent deaths in the United States (*2*,*3*). In 2000, in response to an Institute of Medicine^†^ report noting the need for a national fatal intentional injury surveillance system (*4*), CDC began planning to implement the National Violent Death Reporting System (NVDRS) (*2*). The goals of NVDRS are to

- collect and analyze timely, high-quality data for monitoring the magnitude and characteristics of violent deaths at national, state, and local levels;
- ensure data are disseminated routinely and expeditiously to public health officials, law enforcement officials, policymakers, and the public;
- ensure data are used to develop, implement, and evaluate programs and strategies that are intended to reduce and prevent violent deaths and injuries at national, state, and local levels; and
- build and strengthen partnerships among organizations and communities at national, state, and local levels to ensure that data are collected and used to reduce and prevent violent deaths and injuries.

NVDRS is a state-based active surveillance system that collects data on the characteristics and circumstances associated with violence-related deaths among participating states, the District of Columbia, and Puerto Rico (*2*). Deaths collected by NVDRS include suicides, homicides, legal intervention deaths (i.e., deaths caused by law enforcement acting in the line of duty and other persons with legal authority to use deadly force, excluding legal executions), unintentional firearm deaths, and deaths of undetermined intent that might have been because of violence.^§^ The term “legal intervention” is a classification incorporated into the *International Classification of Diseases, Tenth Revision* (ICD-10) (*5*) and does not denote the lawfulness or legality of the circumstances surrounding a death caused by law enforcement.

Before implementation of NVDRS, single data sources (e.g., death certificates) provided only limited information and few circumstances from which to understand patterns of violent deaths. NVDRS filled this surveillance gap by providing more detailed information. NVDRS is the first system to 1) provide detailed information on circumstances precipitating violent deaths, 2) link multiple source documents so that each incident can contribute to the study of patterns of violent deaths, and 3) link multiple deaths that are related to one another (e.g., multiple homicides, suicide pacts, or homicide followed by suicide of the suspect).

NVDRS data collection began in 2003 with six participating states (Maryland, Massachusetts, New Jersey, Oregon, South Carolina, and Virginia) and has expanded incrementally over time (Figure). Since 2018, CDC has provided NVDRS funding to all 50 states, the District of Columbia, and Puerto Rico. NVDRS data are updated annually and are available to the public through WISQARS* at https://www.cdc.gov/injury/wisqars/nvdrs.html. Case-level NVDRS data are available to interested researchers who meet eligibility requirements via the NVDRS Restricted Access Database (https://www.cdc.gov/violenceprevention/datasources/nvdrs/dataaccess.html).

**FIGURE:**
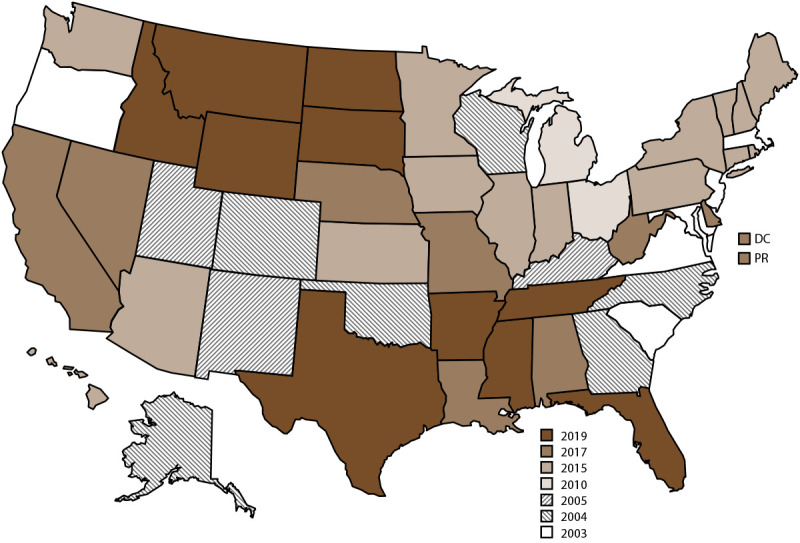
States* and jurisdictions participating in the National Violent Death Reporting System, by year of initial data collection^†^ — United States and Puerto Rico, 2003–2020 **Abbreviations:** DC = District of Columbia; NVDRS = National Violent Death Reporting System; PR = Puerto Rico. * Data for Florida and Hawaii were ineligible to be included in this report because data did not meet the completeness threshold for circumstances. ^†^ Map of United States indicates the year in which the state or jurisdiction began collecting data in NVDRS. Beginning in 2019, all 50 U.S. states, the District of Columbia, and Puerto Rico were participating in the system. California began collecting data for a subset of violent deaths in 2005 but ended data collection in 2009. In 2017, California collected data from death certificates for all NVDRS cases in the state; data for violent deaths that occurred in four counties (Los Angeles, Sacramento, Shasta, and Siskiyou) also include information from coroner or medical examiner reports and law enforcement reports. In 2018, California collected data from death certificates for all violent deaths in the state in 2018 (n = 6,641); data for violent deaths that occurred in 21 counties (Amador, Butte, Fresno, Humboldt, Imperial, Kern, Kings, Lake, Los Angeles, Marin, Mono, Placer, Sacramento, San Benito, San Mateo, San Diego, San Francisco, Shasta, Siskiyou, Ventura, and Yolo) also included information from coroner or medical examiner reports and law enforcement (n = 3,658; 55.1%). In 2019, California collected data from death certificates for all violent deaths in the state in 2019 (n = 6,586); data for violent deaths that occurred in 30 counties (Amador, Butte, Colusa, Fresno, Glenn, Humboldt, Imperial, Kern, Kings, Lassen, Lake, Los Angeles, Marin, Modoc, Mono, Orange, Placer, Sacramento, San Benito, San Francisco, San Mateo, Santa Cruz, Shasta, Siskiyou, Solano, Sonoma, Tehama, Trinity, Ventura, and Yolo) also included information from coroner or medical examiner reports and law enforcement reports (n = 3,645; 55.3%). In 2020, California collected data from death certificates for all violent deaths in the state (n = 6,863); data for violent deaths that occurred in 35 counties (Amador, Butte, Colusa, Contra Costa, Fresno, Glenn, Humboldt, Imperial, Kern, Kings, Lassen, Lake, Los Angeles, Marin, Mendocino, Merced, Modoc, Mono, Orange, Placer, Sacramento, San Benito, San Diego, San Francisco, San Mateo, Santa Cruz, Shasta, Siskiyou, Solano, Sonoma, Stanislaus, Tehama, Trinity, Ventura, and Yolo) also included information from coroner or medical examiner reports and law enforcement reports (n = 4,675; 68.1%). Michigan collected data for a subset of violent deaths during 2010–2013 and collected statewide data beginning in 2014. In 2016, Illinois, Pennsylvania, and Washington began collecting data on violent deaths in a subset of counties that represented at least 80% of all violent deaths in their state or in counties where at least 1,800 violent deaths occurred. Illinois’s 2018 data are for violent deaths that occurred in 28 counties (Adams, Boone, Champaign, Cook, DuPage, Effingham, Fulton, Kane, Kankakee, Kendall, Lake, Lasalle, Livingston, Logan, Macoupin, McDonough, McHenry, McLean, Madison, Peoria, Perry, Rock Island, St. Clair, Sangamon, Tazewell, Vermillion, Will, and Winnebago). Pennsylvania’s 2018 data are for deaths that occurred in 39 counties (Adams, Allegheny, Armstrong, Beaver, Berks, Blair, Bradford, Bucks, Cambria, Carbon, Centre, Chester, Clarion, Clearfield, Clinton, Columbia, Crawford, Dauphin, Delaware, Fayette, Forest, Greene, Indiana, Jefferson, Lackawanna, Lancaster, Lehigh, Luzerne, Monroe, Montgomery, Montour, Northampton, Philadelphia, Schuylkill, Union, Wayne, Westmoreland, Wyoming, and York). Illinois’s 2019 data are for violent deaths that occurred in 47 counties (Adams, Alexander, Bond, Boone, Brown, Bureau, Champaign, Clay, Cook, DeKalb, Douglas, DuPage, Effingham, Fayette, Fulton, Grundy, Henry, Iroquois, Jackson, Jefferson, Kane, Kankakee, Kendall, Lake, Lasalle, Livingston, Logan, Macoupin, McDonough, McHenry, McLean, Madison, Menard, Peoria, Perry, Piatt, Putnam, Rock Island, St. Clair, Sangamon, Schuyler, Stark, Tazewell, Vermilion, Wayne, Will, and Winn). Pennsylvania’s 2019 data are for violent deaths that occurred in 40 counties (Adams, Allegheny, Armstrong, Berks, Blair, Bradford, Bucks, Cameron, Cambria, Carbon, Centre, Chester, Clarion, Clearfield, Clinton, Crawford, Dauphin, Delaware, Erie, Fayette, Forest, Greene, Indiana, Jefferson, Lackawanna, Lancaster, Lehigh, Luzerne, Monroe, Montgomery, Northampton, Philadelphia, Schuylkill, Somerset, Sullivan, Susquehanna, Union, Westmoreland, Wyoming, and York). Washington began collecting statewide data for all violent deaths that occurred beginning in 2018, and Illinois and Pennsylvania began collecting statewide data beginning in 2020. In 2020, Texas collected data from death certificates for all violent deaths in the state in 2020 (n = 6,564); data for violent deaths that occurred in four counties (Bexar, Dallas, Harris, and Tarrant) also included information from coroner or medical examiner reports and law enforcement (n = 2,737 [41.7%]).

This report summarizes NVDRS data on violent deaths that occurred in 48 states, the District of Columbia, and Puerto Rico in 2020. Forty-six states collected statewide data (Alabama, Alaska, Arizona, Arkansas, Colorado, Connecticut, Delaware, Georgia, Idaho, Illinois, Indiana, Iowa, Kansas, Kentucky, Louisiana, Maine, Maryland, Massachusetts, Michigan, Minnesota, Mississippi, Missouri, Montana, Nebraska, Nevada, New Hampshire, New Jersey, New Mexico, New York, North Carolina, North Dakota, Ohio, Oklahoma, Oregon, Pennsylvania, Rhode Island, South Carolina, South Dakota, Tennessee, Utah, Vermont, Virginia, Washington, West Virginia, Wisconsin, and Wyoming). The two remaining states collected data from a subset of counties in their states (35 California counties^¶^ and four Texas counties**). Compared with the 2019 NVDRS report (*6*), this 2020 report includes data for six additional states that met inclusion criteria in 2020 (Arkansas, Idaho, Mississippi, South Dakota, Tennessee, and Texas). Data for Florida and Hawaii were ineligible to be included in this report because the data did not meet the completeness threshold for circumstances (see Inclusion Criteria).

## Methods

NVDRS compiles information from three required data sources: death certificates, coroner and medical examiner records, and law enforcement reports (*2*). Certain participating Violent Death Reporting System (VDRS) programs might also collect information from secondary data sources (e.g., child fatality review team data, Federal Bureau of Investigation Supplementary Homicide Reports, or crime laboratory data). NVDRS combines information for each death and links deaths that are related (e.g., multiple homicides, homicide followed by suicide, or multiple suicides) into a single incident. The ability to analyze linked data can provide a more comprehensive understanding of violent deaths. Participating VDRS programs use vital statistics death certificate files or coroner or medical examiner records to identify violent deaths meeting the NVDRS case definition (see Manner of Death). Each VDRS program reports violent deaths of residents that occurred within the state, district, or territory (i.e., resident deaths) and those of nonresidents for whom a fatal injury occurred within the state, district, or territory (i.e., occurrent deaths). When a violent death is identified, NVDRS data abstractors link source documents, link deaths within each incident, code data elements, and write brief narratives of the incident.

In NVDRS, a violent death is defined as a death resulting from the intentional use of physical force or power, threatened or actual, against oneself, another person, or a group or community (*2*). NVDRS collects information on five manners of death: 1) suicide, 2) homicide, 3) legal intervention death, 4) unintentional firearm death, and 5) death of undetermined intent that might have been because of violence (see Manner of Death). NVDRS cases are determined based on ICD-10 cause of death codes (*5*) or the manner of death assigned by a coroner, medical examiner, or law enforcement officer. Cases are included if they are assigned ICD-10 cause of death codes (Box 1) or a manner of death specified in at least one of the three primary data sources consistent with NVDRS case definitions.

BOX 1 **International Classification of Diseases, Tenth Revision codes used in the National Violent Death Reporting System, 2020**

**Table:** 

Manner of death	Death ≤1 year after injury	Death >1 year after injury	Death any time after injury
Intentional self-harm (suicide)	X60–X84	Y87.0	U03 (attributable to terrorism)
Assault (homicide)	X85–X99, Y00–Y09	Y87.1	U01, U02 (attributable to terrorism)
Event of undetermined intent	Y10–Y34	Y87.2, Y89.9	Not applicable
Unintentional exposure to inanimate mechanical forces (firearms)	W32–W34	Y86	Not applicable
Legal intervention (excluding executions, Y35.5)	Y35.0–Y35.4, Y35.6, Y35.7	Y89.0	Not applicable

NVDRS is an incident-based system, and all decedents associated with a given incident are grouped in one record. Decisions about whether two or more deaths are related and belong to the same incident are made based on the timing of the injuries rather than on the timing of the deaths. Deaths resulting from injuries that are clearly linked by source documents and occur within 24 hours of each other (see Manner of Death) are considered part of the same incident. Examples of an incident include 1) a single isolated violent death, 2) two or more related homicides (including legal intervention deaths) in which the fatal injuries were inflicted <24 hours apart, 3) two or more related suicides or deaths of undetermined intent in which the fatal injuries were inflicted <24 hours apart, and 4) a homicide followed by a suicide in which both fatal injuries were inflicted <24 hours apart (*7*).

Information collected from each data source is entered into the NVDRS web-based system (*2*). This system streamlines data abstraction by allowing abstractors to enter data from multiple sources into the same incident record. Internal validation checks, hover-over features that define selected fields, and other quality control measures also are included within the system. Primacy rules and hierarchal algorithms related to the source documents occur at the local VDRS program level. CDC provides access to the web-based system to each VDRS program. VDRS program personnel are provided ongoing training to learn and adhere to CDC guidance regarding the coding of all variables and technical assistance to help increase data quality. Information abstracted into the system is deidentified at the local VDRS program level, and data are transmitted continuously via the web to a CDC-based server. This activity was reviewed by CDC and was conducted consistent with applicable federal law and CDC policy.^††^

### Manner of Death

A manner (i.e., intent) of death for each decedent is assigned by a trained abstractor who integrates information from all source documents. The abstractor-assigned manner of death must be consistent with at least one required data source; typically, all source documents are consistent regarding the manner of death. When a discrepancy exists, the abstractor must assign a manner of death on the basis of a preponderance of evidence in the source documents; however, such occurrences are rare (*7*). For example, if two sources report a death as a suicide and a third reports it as a death of undetermined intent, the death is coded as a suicide.

NVDRS data are categorized into five abstractor-assigned manners of death: 1) suicide, 2) homicide, 3) legal intervention death, 4) unintentional firearm death, and 5) death of undetermined intent. The case definitions for each manner of death are described as follows:

- **Suicide.** A suicide is a death among persons aged ≥10 years resulting from the use of force against oneself when a preponderance of evidence indicates that the use of force was intentional. The age limit of ≥10 years was established because determining suicide intent in young children can be difficult (*8*). This category also includes the following scenarios: 1) deaths of persons who intended only to injure themselves rather than die by suicide; 2) persons who initially intended to die by suicide and changed their minds but still died as a result of the act; 3) deaths associated with risk-taking behavior without clear intent to inflict a fatal self-injury but associated with high risk for death (e.g., participating in Russian roulette); 4) suicides that occurred while under the influence of substances taken voluntarily; 5) suicides among decedents with mental health problems that affected their thinking, feelings, or mood (e.g., while experiencing an acute episode of a mental health condition, such as schizophrenia or other psychotic conditions, depression, or posttraumatic stress disorder); and 6) suicides involving another person who provided passive (only) assistance to the decedent (e.g., supplying the means or information needed to complete the act). This category does not include deaths caused by chronic or acute substance use without the intent to die, deaths attributed to autoerotic behavior (e.g., self-strangulation during sexual activity), or assisted suicides (legal or nonlegal). Corresponding ICD-10 codes included in NVDRS are X60–X84, Y87.0, and U03 (Box 1).
- **Homicide.** A homicide is a death resulting from the use of physical force or power, threatened or actual, against another person, group, or community when a preponderance of evidence indicates that the use of force was intentional. Two special scenarios that CDC’s National Center for Health Statistics (NCHS) regards as homicides are included in the NVDRS case definition: 1) arson with no specified intent to injure someone and 2) a stabbing with intent unspecified. This category also includes the following scenarios: 1) deaths when the suspect intended to only injure rather than kill the victim, 2) deaths resulting from a heart attack induced when the suspect used force or power against the victim, 3) deaths that occurred when a person killed an attacker in self-defense, 4) deaths resulting from a weapon that discharged unintentionally while being used to control or frighten the victim, 5) deaths attributed to child abuse without intent being specified, 6) deaths attributed to an intentional act of neglect by one person against another, 7) deaths of liveborn infants that resulted from a direct injury because of violence sustained before birth, and 8) deaths identified as a justifiable homicide when the person committing homicide was not a law enforcement officer. This category excludes vehicular homicide without intent to injure, unintentional poisoning deaths because of illicit or prescription drug overdose even when the person who provided drugs was charged with homicide, unintentional firearm deaths (a separate category in NVDRS), combat deaths or acts of war, deaths of unborn fetuses, and deaths of infants that resulted indirectly from violence sustained by the mother before birth (e.g., death from prematurity after premature labor brought on by violence). Corresponding ICD-10 codes included in NVDRS are X85–X99, Y00–Y09, Y87.1, and U01–U02 (Box 1).
- **Legal intervention.** A death from legal intervention is a death in which a person is killed or died as a result of injuries inflicted by a law enforcement officer or another peace officer (i.e., a person with specified legal authority to use deadly force), including military police, while acting in the line of duty. The term “legal intervention” is a classification from ICD-10 (Y35.0) and does not denote the lawfulness or legality of the circumstances surrounding a death caused by law enforcement. Legal intervention deaths also include a small subset of cases in which force was applied without clear lethal intent (e.g., during restraint or when applying force with a typically nondeadly weapon, such as a Taser) or in which the death occurred while the person was fleeing capture. This category excludes legal executions. Corresponding ICD-10 codes included in NVDRS are Y35.0–Y35.4, Y35.6, Y35.7, and Y89.0 (Box 1).
- **Unintentional firearm.** An unintentional firearm death is a death resulting from a penetrating injury or gunshot wound from a weapon that uses a powder charge to fire a projectile and for which a preponderance of evidence indicates that the shooting was not directed intentionally at the decedent with an intent to injure. Examples include the following: 1) a person who received a self-inflicted wound while playing with a firearm; 2) a person who mistakenly believed a gun was unloaded and shot another person; 3) a child aged <6 years who shot himself or herself or another person; 4) a person who died as a result of a celebratory firing that was not intended to frighten, control, or harm anyone; 5) a person who unintentionally shot himself or herself when using a firearm to frighten, control, or harm another person; 6) a soldier who was shot during a field exercise but not in a combat situation; and 7) an infant who died after birth from an unintentional firearm injury that was sustained in utero. This category excludes injuries caused by unintentionally striking a person with the firearm (e.g., hitting a person on the head with the firearm rather than firing a projectile) and unintentional injuries from nonpowder guns (e.g., BB, pellet, or other compressed-air–powered or compressed-gas–powered guns). Corresponding ICD-10 codes included in NVDRS are W32–W34 and Y86 (Box 1).
- **Undetermined intent.** A death of undetermined intent is a death resulting from the use of force or power against oneself or another person for which the evidence indicating one manner of death is no more compelling than evidence indicating another. This category includes coroner or medical examiner rulings in which records from data providers indicate that investigators did not find enough evidence to determine whether the injury was intentional (e.g., unclear whether a drug overdose was unintentional or a suicide). Corresponding ICD-10 codes included in NVDRS are Y10–Y34, Y87.2, and Y89.9 (Box 1).

### Variables Analyzed

NVDRS collects up to approximately 600 unique variables for each death (Boxes 1, 2, and 3). The number of variables recorded for each incident depends on the content and completeness of the source documents. Variables in NVDRS include:

BOX 2 **Methods used to inflict injury — National Violent Death Reporting System, 2020**

- Firearm: method that uses a powder charge to fire a projectile from the weapon (excludes BB gun, pellet gun, or compressed air or gas-powered gun)
- Hanging, strangulation, or suffocation (e.g., hanging by the neck, manual strangulation, or plastic bag over the head)
- Poisoning (e.g., fatal ingestion or injection of an illicit drug, alcohol, pharmaceutical, carbon monoxide, gas, rat poison, or insecticide)
- Sharp instrument (e.g., knife, razor, machete, or pointed instrument)
- Blunt instrument (e.g., club, bat, rock, or brick)
- Fall: being pushed or jumping
- Motor vehicle (e.g., car, bus, motorcycle, or other transport vehicle)
- Personal weapons (e.g., hands, fists, or feet)
- Drowning: inhalation of liquid (e.g., in bathtub, lake, or other source of water or liquid)
- Fire or burns: inhalation of smoke or the direct effects of fire or chemical burns
- Shaking (e.g., shaken baby syndrome)
- Intentional neglect: starvation, lack of adequate supervision, or withholding of health care
- Explosive (e.g., bomb, rocket, or grenade)
- Nonpowder gun (e.g., BB, pellet, compressed air or gas-powered guns)
- Other (single method): any method other than those already listed (e.g., electrocution or exposure to environment or weather)
- Unknown: method not reported or not known

BOX 3 **Circumstances preceding fatal injury, by manner of death — National Violent Death Reporting System, 2020**

**All Manners of Death**

*Mental Health and Substance Abuse*

- Alcohol problem: decedent was perceived by self or others to have a problem with, or to be addicted to or dependent on, alcohol.
- Current depressed mood: decedent was perceived by self or others to be feeling depressed at the time of death.
- Current diagnosed mental health problem: decedent was identified as having a mental health disorder or syndrome listed in the Diagnostic and Statistical Manual, Version 5 (DSM-5), with the exception of alcohol and other substance dependence (these are captured in separate variables).
- Current mental health or substance use treatment: decedent was receiving mental health or substance use treatment as evidenced by a current prescription for a psychotropic medication, visit or visits to a mental health professional, or participation in a therapy group or outpatient program within the previous 2 months.
- History of ever being treated for mental health or substance use problem: decedent was identified as having ever received mental health or substance use treatment.
- Other addiction: decedent was perceived by self or others to have an addiction to or dependency on something other than to alcohol or other substance (e.g., gambling or sex).
- Other substance use problem (excludes alcohol): decedent was perceived by self or others to have a problem with, or be addicted to/dependent on, a substance other than alcohol.
- Type of mental health diagnosis: identifies the type of DSM-5 diagnosis reported for the decedent.

*Crime and Criminal Activity*

- Crime in progress: another serious crime was in progress at the time of the incident.
- Nature of crime: the specific type of other crime that occurred during the incident (e.g., sexual assault, gambling, robbery, or drug trafficking).
- Precipitated by another crime: incident occurred as the result of another serious crime.

*Relationship and Life Stressors*

- Argument or conflict: a specific argument or disagreement led to the victim’s death.
- Caretaker abuse or neglect led to death: decedent was experiencing physical, sexual, or psychological abuse; physical (including medical or dental), emotional, or educational neglect; exposure to a violent environment; or inadequate supervision by a caretaker that led to death.
- Exposure to disaster: decedent was exposed to a disaster (e.g., earthquake, bombing, or COVID-19 pandemic).
- Family relationship problem: decedent was experiencing problems with a family member other than an intimate partner.
- History of child abuse or neglect: as a child, decedent had history of physical, sexual, or psychological abuse; physical (including medical or dental), emotional, or educational neglect; exposure to a violent environment, or inadequate supervision by a caretaker.
- Other relationship problem (non-intimate): decedent was experiencing problems with a friend or associate (other than an intimate partner or family member).
- Perpetrator of interpersonal violence during previous month: decedent perpetrated interpersonal violence during the previous month.
- Physical fight (two persons, not a brawl): a physical fight between two persons that resulted in the death of the decedent, who was either involved in the fight, a bystander, or trying to stop the fight.
- Victim of interpersonal violence during previous month: decedent was the target of interpersonal violence during the past month.

*Crisis Circumstances*

- Crisis during previous or upcoming 2 weeks: current crisis or acute precipitating event or events that either occurred during the previous 2 weeks or was impending in the following 2 weeks (e.g., a trial for a criminal offense begins the following week) and appeared to have contributed to the death. Crises typically are associated with specific circumstance variables (e.g., job problem was a crisis, or a financial problem was a crisis).
- Other crisis: a crisis related to a death but not captured by any of the standard circumstances.

**Suicide or Death of Undetermined Intent**

- Disclosed suicidal intent: decedent had recently expressed suicidal feelings to another person with time for that person to intervene.
- Disclosed intent to whom: type of person (e.g., family member or current or former intimate partner) to whom the decedent recently disclosed suicidal thoughts or plans.
- Eviction or loss of home: decedent was experiencing a recent or impending eviction or other loss of housing, or the threat of eviction or loss of housing.
- Financial problem: decedent was experiencing financial problems (e.g., bankruptcy, overwhelming debt, or foreclosure of a home or business).
- History of attempting suicide: decedent had previously attempted suicide before the fatal incident.
- History of suicidal thoughts or plans: decedent had previously expressed suicidal thoughts or plans.
- Intimate partner problem: decedent was experiencing problems with a current or former intimate partner.
- Job problem: decedent was either experiencing a problem at work or was having a problem with joblessness.
- Left a suicide note: decedent left a note, email message, video, or other communication indicating intent to die by suicide.
- Noncriminal legal problem: decedent was facing civil legal problems (e.g., a child custody or civil lawsuit).
- Other death of family member or friend: decedent was distraught over, or reacting to, the non-suicide death of a family member or friend.
- Physical health problem: decedent was experiencing physical health problems (e.g., a recent cancer diagnosis or chronic pain).
- Recent criminal legal problem: decedent was facing criminal legal problems (e.g., recent or impending arrest or upcoming criminal court date).
- School problem: decedent was experiencing a problem related to school (e.g., poor grades, bullying, social exclusion at school, or performance pressures).
- Suicide of family member or friend: decedent was distraught over, or reacting to, the recent suicide of a family member or friend.
- Traumatic anniversary: the incident occurred on or near the anniversary of a traumatic event in the decedent’s life.

**Homicide or Legal Intervention Death**

- Brawl: mutual physical fight involving three or more persons.
- Drive-by shooting: suspect drove near the decedent and fired a weapon while driving.
- Drug involvement: drug dealing, drug trade, or illicit drug use suspected to have played a role in precipitating the incident.
- Gang related: incident resulted from gang activity or gang rivalry; not used if the decedent was a gang member and the death did not appear to result from gang activity.
- Hate crime: decedent was selected intentionally because of his or her actual or perceived gender, religion, sexual orientation, race, ethnicity, or disability.
- Intimate partner violence related: incident is related to conflict between current or former intimate partners; includes the death of an intimate partner or others (e.g., child, parent, friend, or law enforcement officer) killed in an incident that originated in a conflict between intimate partners.
- Jealousy (lovers’ triangle): jealousy or distress over an intimate partner’s relationship or suspected relationship with another person.
- Justifiable self-defense: decedent was killed by a law enforcement officer in the line of duty or by a civilian in legitimate self-defense or in defense of others.
  Mentally ill suspect: suspect’s attack on decedent was believed to be the direct result of a mental health problem (e.g., schizophrenia or other psychotic condition, depression, or posttraumatic stress disorder).
- Mercy killing: decedent wished to die because of a terminal or hopeless disease or condition, and documentation indicates that the decedent wanted to be killed.
- Prostitution: prostitution or related activity that includes prostitutes, pimps, clients, or others involved in such activity.
- Random violence: decedent was killed in a random act of violence (i.e., an act in which the suspect is not concerned with who is being harmed, just that someone is being harmed).
- Stalking: pattern of unwanted harassing or threatening tactics by either the decedent or suspect.
- Victim used a weapon: decedent used a weapon to attack or defend during the course of the incident.
- Victim was a bystander: decedent was not the intended target in the incident (e.g., pedestrian walking past a gang fight).
- Victim was an intervener assisting a crime victim: decedent was attempting to assist a crime victim at the time of the incident (e.g., a child attempts to intervene and is killed while trying to assist a parent who is being assaulted).
- Victim was a police officer on duty: decedent was a law enforcement officer killed in the line of duty.
- Walk-by assault: decedent was killed by a targeted attack (e.g., ambush) where the suspect fled on foot.

**Unintentional Firearm Death**

*Context of Injury*

- Celebratory firing: shooter fired gun in celebratory manner (e.g., firing into the air at midnight on New Year’s Eve).
- Cleaning gun: shooter pulled trigger or gun discharged while cleaning, repairing, assembling, or disassembling gun.
- Hunting: death occurred any time after leaving home for a hunting trip and before returning home from a hunting trip.
- Loading or unloading gun: gun discharged when the shooter was loading or unloading ammunition.
- Playing with gun: shooter was playing with a gun when it discharged.
- Showing gun to others: gun was being shown to another person when it discharged, or the trigger was pulled.
- Target shooting: shooter was aiming for a target and unintentionally hit the decedent; can be at a shooting range or an informal backyard setting (e.g., teenagers shooting at signposts on a fence).
- Other context of injury: shooting occurred during some context other than those already described.

*Mechanism of Injury*

- Bullet ricocheted: bullet ricocheted from its intended target and struck the decedent.
- Gun fired due to defect or malfunction: gun had a defect or malfunctioned as determined by a trained firearm examiner.
- Gun fired while holstering: gun was being replaced or removed from holster or clothing.
- Gun fired while operating safety or lock: shooter unintentionally fired the gun while operating the safety or lock.
- Gun was dropped: gun discharged when it was dropped.
- Gun was mistaken for toy: gun was mistaken for a toy and was fired without the user understanding the danger.
- Thought gun safety was engaged: shooter thought the safety was on and gun would not discharge.
- Thought gun was unloaded: shooter thought the gun was unloaded for other unspecified reason.
- Thought unloaded, magazine disengaged: shooter thought the gun was unloaded because the magazine was disengaged.
- Unintentionally pulled trigger: shooter unintentionally pulled the trigger (e.g., while grabbing the gun or holding it too tightly).
- Other mechanism of injury: shooting occurred as the result of a mechanism not already described.

- manner of death (i.e., the intent to cause death [suicide, homicide, legal intervention, unintentional, and undetermined] of the person on whom a fatal injury was inflicted) (Box 1);
- demographic information (e.g., age, sex, and race and ethnicity) of victims and suspects (if applicable);
- method of injury (i.e., the mechanism used to inflict a fatal injury) (Box 2);
- location, date, and time of injury and death;
- toxicology findings (for decedents who were tested);
- circumstances (i.e., the events that preceded and were identified by investigators as relevant and therefore might have contributed to the infliction of a fatal injury) (Box 3);
- whether the decedent was a victim (i.e., a person who died as a result of a violence-related injury) or both a suspect and a victim (i.e., a person believed to have inflicted a fatal injury on a victim who then was fatally injured, such as the perpetrator of a homicide followed by suicide);
- information about any known suspects (i.e., a person or persons believed to have inflicted a fatal injury on a victim);
- incident (i.e., an occurrence in which one or more persons sustained a fatal injury that was linked to a common event or perpetrated by the same suspect or suspects during a 24-hour period); and
- type of incident (i.e., a combination of the manner of death and whether single or multiple victims were involved in an incident).

### Circumstances Preceding Death

Circumstances preceding death are defined as the precipitating events that contributed to the infliction of a fatal injury (Box 3). Circumstances are reported based on the content of coroner or medical examiner and law enforcement investigative reports. Certain circumstances are coded to a specific manner of death (e.g., “history of suicidal thoughts or plans” is collected for suicides and deaths of undetermined intent); other circumstances are coded across all manners of death (e.g., “ever treated for mental health or substance use problem”). The data abstractor selects from a list of potential circumstances and is required to code all circumstances that are known to relate to each incident. If circumstances are unknown (e.g., a body found in the woods with no other details reported), the data abstractor does not endorse circumstances; these deaths are then excluded from the denominator for circumstance values. If either the coroner or medical examiner report or law enforcement report indicates the presence of a circumstance, then the abstractor endorses the circumstance. For example, if a law enforcement report indicates that a decedent had disclosed thoughts of suicide or an intent to die by suicide, then the circumstance variable “recent disclosure of suicidal thoughts or intent” is endorsed.

Data abstractors draft two incident narratives: one that summarizes the sequence of events of the incident from the perspective of the coroner or medical examiner record and one that summarizes the sequence of events of the incident from the perspective of the law enforcement report. In addition to briefly summarizing the incident (i.e., the who, what, when, where, and why of the incident), the narratives provide supporting information context, and details on circumstances indicated by the data abstractor for understanding the incident, record information and additional detail that cannot be captured elsewhere, and facilitate data quality control checks on the coding of key variables.

### Coding Training and Quality Control

Ongoing coding support for data abstractors is provided by CDC through an electronic help desk, monthly conference calls, annual in-person or virtual meetings that include coding training for data abstractors, and regular technical assistance conference calls with individual VDRS programs. In addition, all data abstractors are invited to participate in monthly coding work group calls. VDRS programs can conduct additional abstractor training workshops and activities at their own discretion, including through the use of NVDRS Data Abstractor eLearn Training Modules. An NVDRS coding manual (*7*) with CDC-issued standard guidance on coding criteria and examples for each data element is provided to each VDRS program and is publicly available (https://www.cdc.gov/violenceprevention/pdf/nvdrs/nvdrsCodingManual.pdf). Software features that enhance coding reliability include automated validation rules and a hover-over feature containing variable-specific information.

Each year, VDRS programs are required to reabstract a subset of cases using multiple abstractors to identify inconsistencies. In addition, each VDRS program’s data quality plan is evaluated by CDC. Before the data are released each year, CDC conducts a quality control analysis that involves the review of multiple variables for data inconsistencies, with special focus on abstractor-assigned variables (e.g., method of injury and manner of death). If CDC finds inconsistencies, the VDRS program is notified and asked for a response or correction. VDRS programs must meet CDC standards for completeness of circumstance data to be included in the national data set. VDRS programs must have circumstance information abstracted from either the coroner or medical examiner record or the law enforcement report for at least 50% of deaths. However, VDRS programs often exceed this requirement. For 2020, a total of 78.9% of suicides, homicides, and legal intervention deaths in NVDRS had circumstance data from either the coroner or medical examiner record or the law enforcement report. In addition, core variables that represent demographic characteristics (e.g., age, sex, and race and ethnicity) and manners of death were missing or unknown for <0.1% of cases. To ensure the final data set has no duplicate records, during the data closeout process, NVDRS first identifies any records within VDRS programs that match on a subset of 14 key variables and then asks VDRS programs to review these records to determine whether they are true duplicates. One record in any set of two or more records that are true duplicates is retained, and the others are deleted by the VDRS program. Next, NVDRS uses SAS (version 9.4; SAS Institute) to search for any instances of duplicates of a unique identification variable associated with each decedent record. As a third and final check for duplicates, the SAS data set is created with an index that only executes successfully if no duplicates of this identification variable are found.

### Time Frame

VDRS programs are required to begin entering each death into the web-based system within 4 months from the date the violent death occurred. VDRS programs then have an additional 16 months from the end of the calendar year in which the violent death occurred to complete each incident record. Although VDRS programs typically meet timeliness requirements, additional details about an incident occasionally arrive after a deadline has passed. New incidents also might be identified after the deadline (e.g., when a death certificate is revised, new evidence is obtained that changes a manner of death, or an ICD-10 misclassification is corrected to meet the NVDRS case definition). These additional data are incorporated on an ongoing basis into NVDRS when analysis files are updated in real time in the web-based system; 5 months after the 16-month data collection period for the 2020 data year, case counts increased by <0.1%.

### Inclusion Criteria

The inclusion criteria for violent deaths in this report are as follows: 1) cases met the NVDRS case definition for violent death; 2) cases occurred in participating VDRS states, the District of Columbia, or Puerto Rico in 2020; and 3) at least 50% of cases for each included state, district, territory, or subset of counties had circumstance information collected from the coroner or medical examiner record or law enforcement report. Data for Florida and Hawaii were ineligible to be included in this report because data did not meet the completeness threshold for circumstances.

Of the participating VDRS programs, 46 states (Alabama, Alaska, Arizona, Arkansas, Colorado, Connecticut, Delaware, Georgia, Idaho, Illinois, Indiana, Iowa, Kansas, Kentucky, Louisiana, Maine, Maryland, Massachusetts, Michigan, Minnesota, Mississippi, Missouri, Montana, Nebraska, Nevada, New Hampshire, New Jersey, New Mexico, New York, North Carolina, North Dakota, Ohio, Oklahoma, Oregon, Pennsylvania, Rhode Island, South Carolina, South Dakota, Tennessee, Utah, Vermont, Virginia, Washington, West Virginia, Wisconsin, and Wyoming) collected information on all violent deaths that occurred in their state in 2020. In addition, data were collected on all violent deaths that occurred in the District of Columbia and Puerto Rico in 2020. Two states, California and Texas, joined NVDRS with plans to collect data on violent deaths in a subset of counties. California collected data from death certificates for all violent deaths in the state in 2020 (n = 6,902); data for violent deaths that occurred in 35 counties (Amador, Butte, Colusa, Contra Costa, Fresno, Glenn, Humboldt, Imperial, Kern, Kings, Lassen, Lake, Los Angeles, Marin, Mendocino, Merced, Modoc, Mono, Orange, Placer, Sacramento, San Benito, San Diego, San Francisco, San Mateo, Santa Cruz, Shasta, Siskiyou, Solano, Sonoma, Stanislaus, Tehama, Trinity, Ventura, and Yolo) also included information from coroner or medical examiner records and law enforcement reports and are included throughout the rest of the report (n = 4,672; 67.7%). These 35 counties represented 71% of California’s population (*9*). Texas also collected data from death certificates for all violent deaths in the state in 2020 (n = 6,564); data for violent deaths that occurred in four counties (Bexar, Dallas, Harris, and Tarrant) also included information from coroner or medical examiner records and law enforcement reports and are included throughout the rest of the report (n = 2,737; 41.7%). These four counties represented 39% of the state’s population (*9*). Because <100% of violent deaths were abstracted, data from California and Texas do not represent all violent deaths occurring in these states.

### Analyses

This report includes data for violent deaths that occurred in 48 states (46 states collecting statewide data, 35 California counties, and four Texas counties), the District of Columbia, and Puerto Rico in 2020. VDRS program-level data received by CDC as of May 2, 2022, were consolidated and analyzed. The numbers, percentages, and crude rates are presented in aggregate for all deaths by the abstractor-assigned manner of death. The suicide rate was calculated using denominators among populations aged ≥10 years. The rates for other manners of death used denominators among populations of all ages. The rates for cells with frequency <20 are not reported because of the instability of those rates. Denominators for the rates for the two states that did not collect statewide data (California and Texas) correspond to the populations of the counties from which data were collected. The rates could not be calculated for certain variables (e.g., circumstances) because denominators were unknown.

Bridged race 2020 population estimates were used as denominators in the crude rate calculations for the 48 states (46 states collecting statewide data, 35 California counties, and four Texas counties), and the District of Columbia (*10*). For compatible numerators for the rate calculations to be derived, records listing multiple races were recoded to a single race, when possible, using race-bridging methods described by NCHS (https://www.cdc.gov/nchs/nvss/bridged_race.htm) (*11*). Data for Puerto Rico were analyzed separately, as the rates specific to race and ethnicity are not available for Puerto Rico because the Census Bureau estimates for Puerto Rico do not include race or Hispanic or Latino (Hispanic) origin (*12*). Population estimates by sex and age were used as denominators in the crude rate calculations for Puerto Rico (*13*).

## Results

### Violent Deaths in 48 States and the District of Columbia

For 2020, a total of 48 NVDRS states (46 states collecting statewide data, 35 California counties, and four Texas counties) and the District of Columbia collected data on 64,388 incidents involving 66,017 deaths (Supplementary Table S1, https://stacks.cdc.gov/view/cdc/127523). Suicides (n = 38,529; 58.4%) accounted for the highest rate of violent death captured by NVDRS (15.8 per 100,000 population aged ≥10 years). The homicide rate was 7.5 per 100,000 population (n = 20,681; 31.3%). Deaths of undetermined intent (n = 5,429; 8.2%), legal intervention deaths (n = 874; 1.3%), and unintentional firearm deaths (n = 504; <1.0%) occurred at lower rates (2.0, 0.3, and 0.2 per 100,000 population, respectively). Data for deaths by manner that include statewide counts and the rates for California and Texas are available (Supplementary Table S2, https://stacks.cdc.gov/view/cdc/127523).

### Suicides

#### Sex, Age Group, and Race and Ethnicity

For 2020, a total of 48 NVDRS states (46 states collecting statewide data, 35 California counties, and four Texas counties) and the District of Columbia collected data on 38,481 incidents involving 38,529 suicide deaths among persons aged ≥10 years (Supplementary Table S1, https://stacks.cdc.gov/view/cdc/127523). The overall suicide rate was 15.8 per 100,000 population aged ≥10 years (Table 1).

**TABLE 1:** Number, percentage,* and rate^†^ of suicide among persons aged ≥10 years,^§^ by selected demographic characteristics of decedent, method of injury used, and location in which injury occurred — National Violent Death Reporting System, 48 states^¶^ and the District of Columbia, 2020

Characteristic	Male**		Female**		Total	
	No. (%)	Rate	No. (%)	Rate	No. (%)	Rate
**Age group, yrs**						
10–14	325 (1.1)	3.7	165 (2.1)	1.9	**490 (1.3)**	**2.8**
15–19	1,434 (4.7)	15.9	433 (5.5)	5.0	**1,867 (4.8)**	**10.6**
20–24	2,684 (8.7)	28.9	587 (7.5)	6.6	**3,272 (8.5)**	**18.0**
25–29	2,894 (9.4)	28.9	665 (8.5)	6.9	**3,559 (9.2)**	**18.1**
30–34	2,945 (9.6)	30.3	652 (8.3)	6.9	**3,597 (9.3)**	**18.7**
35–44	4,847 (15.8)	27.5	1,324 (16.9)	7.5	**6,171 (16.0)**	**17.5**
45–54	4,700 (15.3)	28.1	1,459 (18.7)	8.5	**6,160 (16.0)**	**18.2**
55–64	4,642 (15.1)	26.8	1,326 (17.0)	7.2	**5,968 (15.5)**	**16.6**
65–74	3,078 (10.0)	24.1	798 (10.2)	5.5	**3,876 (10.1)**	**14.2**
75–84	2,142 (7.0)	36.0	309 (4.0)	4.0	**2,451 (6.4)**	**18.0**
≥85	1,013 (3.3)	51.3	99 (1.3)	2.8	**1,112 (2.9)**	**20.1**
Unknown	3 (<1.0)	—^††^	1 (<1.0)	—	**6 (<1.0)**	**—**
**Race and ethnicity^§§^**						
American Indian or Alaska Native	524 (1.7)	48.9	165 (2.1)	14.6	**689 (1.8)**	**31.2**
Asian or Pacific Islander	856 (2.8)	12.2	359 (4.6)	4.6	**1,215 (3.2)**	**8.2**
Black or African American	2,432 (7.9)	15.9	573 (7.3)	3.4	**3,005 (7.8)**	**9.3**
White	24,141 (78.6)	31.2	6,080 (77.8)	7.6	**30,223 (78.4)**	**19.2**
Hispanic or Latino	2,617 (8.5)	14.2	609 (7.8)	3.4	**3,226 (8.4)**	**8.8**
Other	86 (<1.0)	—	19 (<1.0)	—	**107 (<1.0)**	**—**
Unknown	51 (<1.0)	—	13 (<1.0)	—	**64 (<1.0)**	**—**
**Method of injury**						
Firearm	17,401 (56.7)	14.6	2,491 (31.9)	2.0	**19,892 (51.6)**	**8.2**
Hanging, strangulation, or suffocation	8,200 (26.7)	6.9	2,297 (29.4)	1.9	**10,497 (27.2)**	**4.3**
Poisoning	2,158 (7.0)	1.8	2,135 (27.3)	1.7	**4,294 (11.1)**	**1.8**
Fall	749 (2.4)	0.6	233 (3.0)	0.2	**982 (2.5)**	**0.4**
Sharp instrument	676 (2.2)	0.6	145 (1.9)	0.1	**821 (2.1)**	**0.3**
Motor vehicles (e.g., buses, motorcycles, or other transport vehicles)	462 (1.5)	0.4	144 (1.8)	0.1	**606 (1.6)**	**0.3**
Drowning	250 (<1.0)	0.2	124 (1.6)	0.1	**374 (<1.0)**	**0.2**
Fire or burns	102 (<1.0)	<0.1	37 (<1.0)	<0.1	**139 (<1.0)**	**<0.1**
Blunt instrument	46 (<1.0)	<0.1	11 (<1.0)	—	**57 (<1.0)**	**<0.1**
Other (e.g., Taser, electrocution, nail gun, intentional neglect, or personal weapons)	40 (<1.0)	—	15 (<1.0)	—	**55 (<1.0)**	**—**
Unknown	623 (2.0)	—	186 (2.4)	—	**812 (2.1)**	**—**
**Location of injury**						
House or apartment	21,570 (70.2)	18.1	5,961 (76.2)	4.8	**27,532 (71.5)**	**11.3**
Motor vehicle	1,561 (5.1)	1.3	314 (4.0)	0.3	**1,875 (4.9)**	**0.8**
Natural area	1,421 (4.6)	1.2	236 (3.0)	0.2	**1,658 (4.3)**	**0.7**
Street or highway	868 (2.8)	0.7	142 (1.8)	0.1	**1,010 (2.6)**	**0.4**
Hotel or motel	627 (2.0)	0.5	230 (2.9)	0.2	**857 (2.2)**	**0.4**
Parking lot, public garage, or public transport	519 (1.7)	0.4	87 (1.1)	<0.1	**606 (1.6)**	**0.3**
Jail or prison	550 (1.8)	0.5	53 (<1.0)	<0.1	**603 (1.6)**	**0.3**
Park, playground, or sports or athletic area	447 (1.5)	0.4	89 (1.1)	<0.1	**536 (1.4)**	**0.2**
Bridge	271 (<1.0)	0.2	62 (<1.0)	<0.1	**333 (<1.0)**	**0.1**
Commercial or retail area	254 (<1.0)	0.2	28 (<1.0)	<0.1	**282 (<1.0)**	**0.1**
Railroad tracks	197 (<1.0)	0.2	57 (<1.0)	<0.1	**254 (<1.0)**	**0.1**
Supervised residential facility	165 (<1.0)	0.1	54 (<1.0)	<0.1	**219 (<1.0)**	**<0.1**
Hospital or medical facility	124 (<1.0)	0.1	28 (<1.0)	<0.1	**152 (<1.0)**	**<0.1**
Industrial or construction area	113 (<1.0)	<0.1	10 (<1.0)	—	**123 (<1.0)**	**<0.1**
Farm	95 (<1.0)	<0.1	10 (<1.0)	—	**105 (<1.0)**	**<0.1**
Cemetery, graveyard, or other burial ground	82 (<1.0)	<0.1	19 (<1.0)	—	**101 (<1.0)**	**<0.1**
Other location^¶¶^	667 (2.2)	—	85 (1.1)	—	**752 (2.0)**	**—**
Unknown	1,176 (3.8)	—	353 (4.5)	—	**1,531 (4.0)**	**—**
**Total**	**30,707 (100)**	**25.7**	**7,818 (100)**	**6.3**	**38,529 (100)**	**15.8**

* Percentages might not total 100% because of rounding.

^†^ Per 100,000 population.

^§^ Suicide is not reported for decedents aged <10 years per standard in the suicide prevention literature. Denominators for suicide rates represent the total population aged ≥10 years.

^¶^ Includes all U.S. states, with exception of Florida and Hawaii. Data for California are for violent deaths that occurred in 35 counties (Amador, Butte, Colusa, Contra Costa, Fresno, Glenn, Humboldt, Imperial, Kern, Kings, Lassen, Lake, Los Angeles, Marin, Mendocino, Merced, Modoc, Mono, Orange, Placer, Sacramento, San Benito, San Diego, San Francisco, San Mateo, Santa Cruz, Shasta, Siskiyou, Solano, Sonoma, Stanislaus, Tehama, Trinity, Ventura, and Yolo). Data for Texas are for violent deaths that occurred in four counties (Bexar, Dallas, Harris, and Tarrant). Denominators for the rates for California and Texas represent only the populations of the counties from which the data were collected.

** Sex was unknown for four decedents.

^††^ Dashes indicate cell data are suppressed because number of decedents is <20 or when characteristic response is “Other” or “Unknown.”

^§§^ Persons of Hispanic or Latino (Hispanic) origin might be of any race but were categorized as Hispanic; all racial groups were non-Hispanic.

^¶¶^ Other location includes (in descending order): preschool/school/college/school bus, office building, abandoned house/building/warehouse, synagogue/church/temple, bar/nightclub, or other unspecified location.

The overall suicide rate for males (25.7 per 100,000 population aged ≥10 years) was 4.1 times the rate for females (6.3 per 100,000 population aged ≥10 years) (Table 1). The suicide rate for males ranged from 1.9 to 18.3 times the rate for females across age groups and 2.7 to 4.7 times the rate for females across racial and ethnic groups. Adults aged ≥85 years (20.1 per 100,000 population aged ≥10 years), 30–34 years (18.7 per 100,000 population aged ≥10 years), and 45–54 years (18.2 per 100,000 population aged ≥10 years) had the highest rates of suicide across age groups. White persons accounted for a majority (78.4%) of suicides; however, AI/AN persons had the highest rate of suicide (31.2 per 100,000 population aged ≥10 years) among all racial and ethnic groups.

Among male suicide decedents, nearly one half (46.2%) were aged 35–64 years (Table 1). By age group, men aged ≥85 years had the highest rate of suicide (51.3 per 100,000 population aged ≥10 years), followed by men aged 75–84 years (36.0 per 100,000 population aged ≥10 years) and 30–34 years (30.3 per 100,000 population aged ≥10 years). Across racial and ethnic groups, AI/AN males had the highest rate of suicide (48.9 per 100,000 population aged ≥10 years), followed by White males (31.2 per 100,000 population aged ≥10 years). The rate of suicide for AI/AN males was 4.0 times the rate for males with the lowest rate (i.e., non-Hispanic Asian or Pacific Islander [A/PI]; 12.2 per 100,000 population aged ≥10 years). The suicide rate was 15.9 per 100,000 population aged ≥10 years for Black males and 14.2 per 100,000 population aged ≥10 years for Hispanic males.

Among females, those aged 35–64 years accounted for 52.6% of suicides (Table 1). Females aged 45–54 years had the highest rate of suicide (8.5 per 100,000 population), followed by those aged 35–44 years (7.5 per 100,000 population) and 55–64 years (7.2 per 100,000 population). The suicide rate was highest among AI/AN females (14.6 per 100,000 population aged ≥10 years), followed by White (7.6 per 100,000 population aged ≥10 years), A/PI (4.6 per 100,000 population aged ≥10 years), and Black and Hispanic (both 3.4 per 100,000 population aged ≥10 years) females. The suicide rate for AI/AN females was 4.3 times the rate for females with the lowest rates (i.e., Black and Hispanic females).

#### Method and Location of Injury

A firearm was used in more than one half (51.6%; 8.2 per 100,000 population aged ≥10 years) of suicides, followed by hanging, strangulation, or suffocation (27.2%; 4.3 per 100,000 population aged ≥10 years) and poisoning (11.1%; 1.8 per 100,000 population aged ≥10 years) (Table 1). Among males, the most common method of injury was a firearm (56.7%), followed by hanging, strangulation, or suffocation (26.7%). Among females, firearm (31.9%) was also the most common method of injury, followed by hanging, strangulation, or suffocation (29.4%) and poisoning (27.3%). Among all suicide decedents, the most common location of suicide was a house or apartment (71.5%), followed by a motor vehicle (4.9%), a natural area (4.3%), a street or highway (2.6%), and a hotel or motel (2.2%).

#### Toxicology Results of Decedent

Toxicology tests for blood alcohol concentration (BAC) were conducted for 47.4% of suicide decedents (Table 2). Among those with positive results for alcohol (41.0%), 66.0% had a BAC ≥0.08 g/dL. Tests for the following substances were conducted for the percentage of decedents indicated in parentheses: amphetamines (38.0%), antidepressants (24.4%), barbiturates (31.3%), benzodiazepines (37.0%), cannabis (commonly referred to as marijuana; 34.7%), cocaine (36.9%), and opioids (39.6%). Positive results were found for 17.5% of decedents tested for amphetamines. Among those tested for antidepressants, 36.1% had positive results at the time of death; 2.1% of those tested for barbiturates had positive results, 21.9% of those tested for benzodiazepines had positive results, 28.3% of those tested for cannabis had positive results, and 6.1% of those tested for cocaine had positive results. Test results for opioids (including illicit and prescription opioids) were positive for 22.1% of decedents tested for these substances. Carbon monoxide was tested for a substantially smaller proportion of decedents (4.0%) but was identified in approximately one third of those decedents (35.3%).

**TABLE 2:** Number* and percentage of suicide decedents tested for alcohol and drugs and whose results were positive,^†^ by toxicology — National Violent Death Reporting System, 48 states^§^ and the District of Columbia, 2020

Toxicology	Tested No. (%)	Positive No. (%)
Blood alcohol concentration^¶^	18,245 (47.4)	7,480 (41.0)
Alcohol <0.08 g/dL	N/A	2,046 (27.4)
Alcohol ≥0.08 g/dL	N/A	4,937 (66.0)
Alcohol positive — level unknown	N/A	497 (6.6)
Amphetamines	14,635 (38.0)	2,554 (17.5)
Anticonvulsants	7,479 (19.4)	1,339 (17.9)
Antidepressants	9,390 (24.4)	3,394 (36.1)
Antipsychotics	7,089 (18.4)	914 (12.9)
Barbiturates	12,064 (31.3)	250 (2.1)
Benzodiazepines	14,248 (37.0)	3,116 (21.9)
Cannabis	13,378 (34.7)	3,789 (28.3)
Carbon monoxide	1,540 (4.0)	544 (35.3)
Cocaine	14,235 (36.9)	867 (6.1)
Muscle relaxant	7,632 (19.8)	426 (5.6)
Opioids	15,271 (39.6)	3,370 (22.1)
Other drugs or substances**	2,385 (6.2)	2,250 (94.3)

**Abbreviation:** N/A = not applicable.

* N = 38,529.

^†^ Percentage is of decedents tested for toxicology. Denominator for the percentage positive is the percentage tested.

^§^ Includes all U.S. states, with exception of Florida and Hawaii. Data for California are for violent deaths that occurred in 35 counties (Amador, Butte, Colusa, Contra Costa, Fresno, Glenn, Humboldt, Imperial, Kern, Kings, Lassen, Lake, Los Angeles, Marin, Mendocino, Merced, Modoc, Mono, Orange, Placer, Sacramento, San Benito, San Diego, San Francisco, San Mateo, Santa Cruz, Shasta, Siskiyou, Solano, Sonoma, Stanislaus, Tehama, Trinity, Ventura, and Yolo). Data for Texas are for violent deaths that occurred in four counties (Bexar, Dallas, Harris, and Tarrant).

^¶^ Blood alcohol concentration of ≥0.08 g/dL is over the legal limit in all states and is used as the standard for intoxication.

** Other drugs or substances indicated if any results were positive; levels for these drugs or substances were not measured.

#### Precipitating Circumstances

Circumstances were identified in 32,307 (83.9%) suicides (Table 3). Overall, a mental health problem was the most common circumstance, with approximately one half (47.8%) of decedents having a current diagnosed mental health problem and 31.9% experiencing a depressed mood at the time of death. Among the 15,433 decedents with a current diagnosed mental health problem, depression or dysthymia (73.1%), anxiety disorder (22.5%), and bipolar disorder (15.1%) were the most common diagnoses. Alcohol use problems were reported for 18.4% of suicide decedents, and other substance use problems (unrelated to alcohol) were reported for 17.7% of suicide decedents. Among suicide decedents, 24.1% were receiving mental health or substance use treatment at the time of death and 32.0% had a history of having been treated for a mental health or substance use problem (Table 3).

**TABLE 3:** Number* and percentage^†^ of suicides among persons aged ≥10 years,^§^ by decedent sex and precipitating circumstances — National Violent Death Reporting System, 48 states^¶^ and the District of Columbia, 2020

Precipitating circumstance	Male No. (%)	Female No. (%)	Total No. (%)
**Mental health and substance use**			
Current diagnosed mental health problem**	11,207 (43.8)	4,225 (63.0)	**15,433 (47.8)**
Depression or dysthymia	8,037 (71.7)	3,242 (76.7)	**11,279 (73.1)**
Anxiety disorder	2,291 (20.4)	1,181 (28.0)	**3,473 (22.5)**
Bipolar disorder	1,503 (13.4)	826 (19.6)	**2,330 (15.1)**
Schizophrenia	878 (7.8)	222 (5.3)	**1,100 (7.1)**
Posttraumatic stress disorder	739 (6.6)	195 (4.6)	**934 (6.1)**
Attention deficit disorder or attention deficit hyperactivity disorder	407 (3.6)	86 (2.0)	**493 (3.2)**
Dementia	220 (2.0)	39 (<1.0)	**259 (1.7)**
Autism spectrum	111 (<1.0)	10 (<1.0)	**121 (<1.0)**
Obsessive compulsive disorder	82 (<1.0)	24 (<1.0)	**106 (<1.0)**
Eating disorder	6 (<1.0)	28 (<1.0)	**34 (<1.0)**
Fetal alcohol syndrome	4 (<1.0)	0 (0.0)	**4 (<1.0)**
Down syndrome	0 (0.0)	0 (0.0)	**0 (0.0)**
Other	504 (4.5)	181 (4.3)	**685 (4.4)**
Unknown	855 (7.6)	281 (6.7)	**1,136 (7.4)**
History of ever being treated for a mental health or substance use problem	7,280 (28.4)	3,059 (45.6)	**10,339 (32.0)**
Current depressed mood	8,145 (31.8)	2,166 (32.3)	**10,311 (31.9)**
Current mental health or substance use treatment	5,339 (20.9)	2,435 (36.3)	**7,774 (24.1)**
Alcohol problem	4,893 (19.1)	1,049 (15.6)	**5,943 (18.4)**
Other substance use problem (excludes alcohol)	4,472 (17.5)	1,240 (18.5)	**5,712 (17.7)**
Other addiction (e.g., gambling, or sexual)	224 (<1.0)	51 (<1.0)	**275 (<1.0)**
**Interpersonal**			
Intimate partner problem	6,934 (27.1)	1,661 (24.8)	**8,596 (26.6)**
Family relationship problem	1,915 (7.5)	659 (9.8)	**2,574 (8.0)**
Other death of family member or friend	1,454 (5.7)	448 (6.7)	**1,902 (5.9)**
Other relationship problem (nonintimate)	593 (2.3)	151 (2.3)	**744 (2.3)**
Perpetrator of interpersonal violence during past month	680 (2.7)	57 (<1.0)	**737 (2.3)**
Suicide of family member or friend	542 (2.1)	186 (2.8)	**728 (2.3)**
Victim of interpersonal violence during past month	64 (<1.0)	63 (<1.0)	**127 (<1.0)**
**Life stressor**			
Crisis during previous or upcoming 2 weeks	7,853 (30.7)	1,760 (26.2)	**9,613 (29.8)**
Physical health problem	5,184 (20.3)	1,238 (18.5)	**6,422 (19.9)**
Argument or conflict	4,380 (17.1)	1,121 (16.7)	**5,501 (17.0)**
Job problem	2,437 (9.5)	452 (6.7)	**2,889 (8.9)**
Recent criminal legal problem	2,033 (7.9)	168 (2.5)	**2,201 (6.8)**
Financial problem	1,747 (6.8)	368 (5.5)	**2,115 (6.5)**
Exposure to disaster	1,203 (4.7)	366 (5.5)	**1,569 (4.9)**
Noncriminal legal problem	748 (2.9)	176 (2.6)	**925 (2.9)**
Eviction or loss of home	678 (2.6)	193 (2.9)	**871 (2.7)**
History of child abuse or neglect	242 (<1.0)	172 (2.6)	**414 (1.3)**
School problem	293 (1.1)	106 (1.6)	**399 (1.2)**
Physical fight (two persons, not a brawl)	289 (1.1)	41 (<1.0)	**330 (1.0)**
Traumatic anniversary	165 (<1.0)	67 (<1.0)	**232 (<1.0)**
Caretaker abuse or neglect led to suicide	18 (<1.0)	23 (<1.0)	**41 (<1.0)**
**Crime and criminal activity**			
Precipitated by another crime	1,107 (4.3)	107 (1.6)	**1,214 (3.8)**
Crime in progress^††^	353 (31.9)	25 (23.4)	**378 (31.1)**
**Suicide event**			
History of suicidal thoughts or plans	8,798 (34.4)	2,662 (39.7)	**11,461 (35.5)**
Left a suicide note	7,129 (27.9)	2,464 (36.7)	**9,594 (29.7)**
History of suicide attempts	3,844 (15.0)	2,063 (30.7)	**5,908 (18.3)**
**Suicide disclosure**			
Disclosed suicidal intent	5,979 (23.4)	1,515 (22.6)	**7,494 (23.2)**
Disclosed intent to whom^§§^			
Previous or current intimate partner	2,398 (40.1)	514 (33.9)	**2,912 (38.9)**
Other family member	2,070 (34.6)	538 (35.5)	**2,608 (34.8)**
Friend or colleague	820 (13.7)	252 (16.6)	**1,072 (14.3)**
Health care worker	291 (4.9)	98 (6.5)	**389 (5.2)**
Through social media or other electronic means	271 (4.5)	78 (5.1)	**349 (4.7)**
Neighbor	96 (1.6)	32 (2.1)	**128 (1.7)**
Other	508 (8.5)	110 (7.3)	**618 (8.2)**
Unknown	431 (7.2)	129 (8.5)	**560 (7.5)**
**Total^¶¶^**	**25,597 (83.4)**	**6,709 (85.8)**	**32,307 (83.9)**

* Includes suicides with one or more precipitating circumstances. More than one circumstance could have been present per decedent.

^†^ Denominator includes those suicides with one or more precipitating circumstances. The sums of percentages in columns exceed 100% because more than one circumstance could have been present per decedent.

^§^ Suicide is not reported for decedents aged <10 years as per standard in the suicide prevention literature.

^¶^ Includes all U.S. states, with exception of Florida and Hawaii. Data for California are for violent deaths that occurred in 35 counties (Amador, Butte, Colusa, Contra Costa, Fresno, Glenn, Humboldt, Imperial, Kern, Kings, Lassen, Lake, Los Angeles, Marin, Mendocino, Merced, Modoc, Mono, Orange, Placer, Sacramento, San Benito, San Diego, San Francisco, San Mateo, Santa Cruz, Shasta, Siskiyou, Solano, Sonoma, Stanislaus, Tehama, Trinity, Ventura, and Yolo). Data for Texas are for violent deaths that occurred in four counties (Bexar, Dallas, Harris, and Tarrant).

** Includes decedents with one or more diagnosed current mental health problems; therefore, sums of percentages for the diagnosed conditions exceed 100%. Denominator includes the number of decedents with ≥1 current diagnosed mental health problems.

^††^ Denominator includes those decedents involved in an incident that was precipitated by another crime.

^§§^ Denominator includes decedents who disclosed intent. The sum of percentages exceeds 100% because more than one response could have been present per decedent.

^¶¶^ Circumstances were unknown for 6,222 decedents (5,110 males, 1,109 females, and four unknown); total number of suicide decedents = 38,529 (30,707 males, 7,818 females, and four unknown).

The most commonly reported interpersonal or life stressor–related precipitating circumstances for suicide were a recent or impending crisis during the previous or upcoming 2 weeks (29.8%), intimate partner problem (26.6%), physical health problem (19.9%), and argument or conflict (17.0%) (Table 3) (Supplementary Table S4, https://stacks.cdc.gov/view/cdc/127523). Among other circumstances related to suicide, 35.5% of decedents had a history of suicidal thoughts or plans, 29.7% left a suicide note, 23.2% had disclosed suicidal intent to another person, and 18.3% had a history of attempting suicide. Among those who disclosed intent, the greatest proportion of disclosures were to a previous or current intimate partner (38.9%), followed by a family member other than an intimate partner (34.8%) and friend or colleague (14.3%).

When examining known circumstances by sex, a larger percentage of female decedents (63.0%) had a current diagnosed mental health problem than did male decedents (43.8%) (Table 3). Male and female suicide decedents had similar percentages of depressed mood at the time of their death (31.8% and 32.3%, respectively). A larger percentage of female decedents (36.3%) than male decedents (20.9%) were known to have been receiving mental health or substance treatment at the time of death. Suicide events (e.g., leaving a suicide note), history of suicidal thoughts or plans, and history of attempting suicide occurred more frequently and at higher rates among females than males.

### Homicides

#### Sex, Age Group, and Race and Ethnicity

For 2020, a total of 48 NVDRS states (46 states collecting statewide data, 35 California counties, and four Texas counties) and the District of Columbia collected data on 19,705 incidents (Supplementary Table S1, https://stacks.cdc.gov/view/cdc/127523) involving 20,681 homicide deaths. The overall homicide rate was 7.5 per 100,000 population (Table 4).

**TABLE 4:** Number, percentage,* and rate^†^ of homicides, by selected demographic characteristics of decedent, method of injury used, location in which injury occurred, and victim-to-suspect relationship^§^ — National Violent Death Reporting System, 48 states^¶^ and the District of Columbia, 2020

Characteristic	Male		Female		Total	
	No. (%)	Rate	No. (%)	Rate	No. (%)	Rate
**Age group, yrs**						
<1	136 (<1.0)	8.5	81 (2.1)	5.3	**217 (1.0)**	**6.9**
1–4	151 (<1.0)	2.3	102 (2.6)	1.6	**253 (1.2)**	**1.9**
5–9	69 (<1.0)	0.8	68 (1.7)	0.8	**137 (<1.0)**	**0.8**
10–14	164 (<1.0)	1.9	61 (1.6)	0.7	**225 (1.1)**	**1.3**
15–19	1,876 (11.2)	20.9	273 (7.0)	3.2	**2,149 (10.4)**	**12.2**
20–24	2,865 (17.1)	30.8	457 (11.7)	5.1	**3,322 (16.1)**	**18.2**
25–29	2,806 (16.7)	28.0	481 (12.3)	5.0	**3,287 (15.9)**	**16.7**
30–34	2,334 (13.9)	24.0	413 (10.6)	4.4	**2,747 (13.3)**	**14.3**
35–44	3,035 (18.1)	17.2	682 (17.5)	3.9	**3,717 (18.0)**	**10.5**
45–54	1,634 (9.7)	9.8	492 (12.6)	2.9	**2,126 (10.3)**	**6.3**
55–64	1,092 (6.5)	6.3	379 (9.7)	2.0	**1,471 (7.1)**	**4.1**
65–74	417 (2.5)	3.3	216 (5.5)	1.5	**633 (3.1)**	**2.3**
75–84	146 (<1.0)	2.5	128 (3.3)	1.7	**274 (1.3)**	**2.0**
≥85	51 (<1.0)	2.6	62 (1.6)	1.7	**113 (<1.0)**	**2.0**
Unknown	8 (<1.0)	—**	2 (<1.0)	—	**10 (<1.0)**	**—**
**Race and ethnicity^††^**						
American Indian or Alaska Native	275 (1.6)	21.9	84 (2.2)	6.4	**359 (1.7)**	**14.0**
Asian or Pacific Islander	189 (1.1)	2.3	79 (2.0)	0.9	**268 (1.3)**	**1.6**
Black or African American	10,377 (61.8)	57.6	1,618 (41.5)	8.2	**11,995 (58.0)**	**31.8**
White	3,385 (20.2)	3.9	1,619 (41.5)	1.8	**5,004 (24.2)**	**2.9**
Hispanic or Latino	2,492 (14.8)	11.2	475 (12.2)	2.2	**2,967 (14.3)**	**6.7**
Other	39 (<1.0)	—	13 (<1.0)	—	**52 (<1.0)**	**—**
Unknown	27 (<1.0)	—	9 (<1.0)	—	**36 (<1.0)**	**—**
**Method of injury**						
Firearm	13,440 (80.1)	9.9	2,413 (61.9)	1.7	**15,853 (76.7)**	**5.7**
Sharp instrument	1,371 (8.2)	1.0	508 (13.0)	0.4	**1,879 (9.1)**	**0.7**
Blunt instrument	465 (2.8)	0.3	215 (5.5)	0.2	**680 (3.3)**	**0.3**
Personal weapons (e.g., hands, feet, or fists)	360 (2.1)	0.3	153 (3.9)	0.1	**513 (2.5)**	**0.2**
Hanging, strangulation, or suffocation	142 (<1.0)	0.1	177 (4.5)	0.1	**319 (1.5)**	**0.1**
Motor vehicles (e.g., buses, motorcycles, or other transport vehicles)	111 (<1.0)	<0.1	61 (1.6)	<0.1	**172 (<1.0)**	**<0.1**
Poisoning	50 (<1.0)	<0.1	39 (1.0)	<0.1	**89 (<1.0)**	**<0.1**
Fire or burns	53 (<1.0)	<0.1	36 (<1.0)	<0.1	**89 (<1.0)**	**<0.1**
Intentional neglect	32 (<1.0)	<0.1	33 (<1.0)	<0.1	**65 (<1.0)**	**<0.1**
Fall	26 (<1.0)	<0.1	19 (<1.0)	—	**45 (<1.0)**	**<0.1**
Shaking (e.g., shaken baby syndrome)	17 (<1.0)	—	9 (<1.0)	—	**26 (<1.0)**	**<0.1**
Drowning	13 (<1.0)	—	8 (<1.0)	—	**21 (<1.0)**	**<0.1**
Other (e.g., Taser, electrocution, or nail gun)	25 (<1.0)	—	12 (<1.0)	—	**37 (<1.0)**	**—**
Unknown	679 (4.0)	—	214 (5.5)	—	**893 (4.3)**	**—**
**Location of injury**						
House or apartment	6,146 (36.6)	4.5	2,334 (59.9)	1.7	**8,480 (41.0)**	**3.1**
Street or highway	4,203 (25.0)	3.1	406 (10.4)	0.3	**4,609 (22.3)**	**1.7**
Motor vehicle	1,785 (10.6)	1.3	351 (9.0)	0.3	**2,136 (10.3)**	**0.8**
Parking lot, public garage, or public transport	855 (5.1)	0.6	80 (2.1)	<0.1	**935 (4.5)**	**0.3**
Commercial or retail area	709 (4.2)	0.5	68 (1.7)	<0.1	**777 (3.8)**	**0.3**
Hotel or motel	229 (1.4)	0.2	72 (1.8)	<0.1	**301 (1.5)**	**0.1**
Natural area	232 (1.4)	0.2	66 (1.7)	<0.1	**298 (1.4)**	**0.1**
Park, playground, or sports or athletic area	193 (1.1)	0.1	23 (<1.0)	<0.1	**216 (1.0)**	**<0.1**
Bar or nightclub	178 (1.1)	0.1	20 (<1.0)	<0.1	**198 (<1.0)**	**<0.1**
Jail or prison	123 (<1.0)	<0.1	0 (0.0)	—	**123 (<1.0)**	**<0.1**
Other location^§§^	590 (3.5)	—	127 (3.3)	—	**717 (3.5)**	**—**
Unknown	1,541 (9.2)	—	350 (9.0)	—	**1,891 (9.1)**	**—**
**Relationship of victim to suspect^¶¶^**						
Acquaintance or friend	1,475 (30.9)	1.1	186 (9.2)	0.1	**1,661 (24.5)**	**0.6**
Spouse or intimate partner (current or former)	375 (7.9)	0.3	1,006 (50.0)	0.7	**1,381 (20.4)**	**0.5**
Other person, known to victim	1,060 (22.2)	0.8	169 (8.4)	0.1	**1,229 (18.1)**	**0.4**
Stranger	863 (18.1)	0.6	137 (6.8)	0.1	**1,000 (14.7)**	**0.4**
Other relative	354 (7.4)	0.3	136 (6.8)	0.1	**490 (7.2)**	**0.2**
Child***	262 (5.5)	0.2	169 (8.4)	0.1	**431 (6.4)**	**0.2**
Parent***	187 (3.9)	0.1	167 (8.3)	0.1	**354 (5.2)**	**0.1**
Rival gang member	83 (1.7)	<0.1	6 (<1.0)	—	**89 (1.3)**	**<0.1**
Child of suspect's boyfriend or girlfriend (e.g., child killed by mom's boyfriend)	46 (<1.0)	<0.1	29 (1.4)	<0.1	**75 (1.1)**	**<0.1**
Other relationship^†††^	67 (1.4)	—	6 (<1.0)	—	**73 (1.1)**	**—**
**Total**	**16,784 (100)**	**12.3**	**3,897 (100)**	**2.8**	**20,681 (100)**	**7.5**

* Percentages might not total 100% because of rounding.

^†^ Per 100,000 population.

^§^ The following sentence can be used as a guide for interpreting victim-suspect relationship: “The victim is the [relationship] of the suspect.” For example, when a parent kills a child, the relationship is “Child” not “Parent” (“The victim is the child of the suspect”). Please note that this sentence is intended to be a general guide. However, some relationships might not be captured by this sentence (e.g., other person known to victim or victim was law enforcement officer killed in the line of duty).

^¶^ Includes all U.S. states, with exception of Florida and Hawaii. Data for California are for violent deaths that occurred in 35 counties (Amador, Butte, Colusa, Contra Costa, Fresno, Glenn, Humboldt, Imperial, Kern, Kings, Lassen, Lake, Los Angeles, Marin, Mendocino, Merced, Modoc, Mono, Orange, Placer, Sacramento, San Benito, San Diego, San Francisco, San Mateo, Santa Cruz, Shasta, Siskiyou, Solano, Sonoma, Stanislaus, Tehama, Trinity, Ventura, and Yolo). Data for Texas are for violent deaths that occurred in four counties (Bexar, Dallas, Harris, and Tarrant). Denominators for the rates for California and Texas represent only the populations of the counties from which the data were collected.

** Cell data suppressed because number of decedents <20 or characteristic response is “Other” or “Unknown.”

^††^ Persons of Hispanic or Latino (Hispanic) origin might be of any race but were categorized as Hispanic; all racial groups were non-Hispanic.

^§§^ Other location includes (in descending order): abandoned house/building/warehouse, supervised residential facility, industrial or construction area, hospital or medical facility, office building, synagogue/church/temple, preschool/school/college/school bus, farm, cemetery/graveyard/other burial ground, railroad tracks, bridge, and other unspecified location.

^¶¶^ Percentage is based on the number of homicide decedents with a known victim-to-suspect relationship (n = 6,783 [32.8%]; 4,772 [28.4%] males and 2,011 [51.6%] females); victim-to-suspect relationship was unknown for 13,898 decedents.

*** Includes adoptive family members (e.g., adopted child), stepfamily members (e.g., stepparent), and foster family members (e.g., foster child).

^†††^ Other relationship includes (in descending order): an intimate partner of suspect's parent (e.g., teenager kills his mother’s boyfriend), victim was law enforcement officer injured in line of duty, and victim injured by a law enforcement officer.

The homicide rates were higher among males than females across nearly all age groups, and the rate was highest among adults aged 20–24 years (18.2 per 100,000 population) (Table 4). The homicide rate for men aged 20–24 years (30.8 per 100,000 population) was six times the rate for females in the same age group (5.1 per 100,000 population). Among males, the rate of homicide was highest among adults aged 20–24 years (30.8 per 100,000 population) and 25–29 years (28.0 per 100,000 population). Among females, the rate of homicide was highest among infants (i.e., children aged <1 year; 5.3 per 100,000 population). Among all children who were homicide victims, the overall homicide rate for infants (6.9 per 100,000 population) was 3.6 times the overall rate for children aged 1–4 years (1.9 per 100,000 population) and 8.6 times the rate for children aged 5–9 years (0.8 per 100,000 population).

Black persons accounted for 61.8% of male homicide victims and more than one half (58.0%) of all homicide victims (Table 4). Black males had the highest rate of homicide compared with males in all other racial and ethnic groups (57.6 per 100,000 population); this rate was 25.0 times the rate for A/PI males (2.3 per 100,000 population), 14.8 times the rate for White males (3.9 per 100,000 population), 5.1 times the rate for Hispanic males (11.2 per 100,000 population), and 2.6 times the rate for AI/AN males (21.9 per 100,000 population). Among females, the homicide rate was also highest among Black females (8.2 per 100,000 population) (Table 4), followed by AI/AN females (6.4 per 100,000 population), Hispanic females (2.2 per 100,000 population), White females (1.8 per 100,000 population), and A/PI females (0.9 per 100,000 population).

#### Method, Location of Injury, and Victim-Suspect Relationship

The weapons most commonly used in homicides were firearms, used in 76.7% of homicides overall; followed by a sharp instrument (9.1%); a blunt instrument (3.3%); personal weapons (e.g., hands, feet, or fists; 2.5%); and hanging, strangulation, or suffocation (1.5%) (Table 4). The method was unknown in 4.3% of homicides. A firearm was the most common method of injury for both males (80.1%) and females (61.9%); however, the firearm homicide rate for males (9.9 per 100,000 population) was 5.8 times the rate for females (1.7 per 100,000 population). A larger proportion of homicides among females than males involved a sharp instrument (13.0% versus 8.2%, respectively); blunt instrument (5.5% versus 2.8%, respectively); hanging, strangulation, or suffocation (4.5% versus <1.0%, respectively); and personal weapons (3.9% versus 2.1%, respectively). Among all homicide victims, a house or apartment was the most common location of homicide (41.0%); followed by a street or highway (22.3%); a motor vehicle (10.3%); and a parking lot, public garage, or public transport (4.5%). A larger proportion of homicides among females (59.9%) than among males (36.6%) occurred at a house or apartment, whereas a larger proportion of homicides among males (25.0%) than among females (10.4%) occurred on a street or highway.

The relationship of the victim to the suspect was known for 32.8% of homicides (28.4% of males and 51.6% of females) (Table 4). For males, when the relationship was known, the victim-suspect relationship was most often an acquaintance or friend (30.9%); other person known to the victim, but the exact nature of the relationship was unclear (22.2%); a stranger (18.1%); a current or former intimate partner (7.9%); or other relative (7.4%). For females, when the relationship was known, one half (50.0%) of suspects were a current or former intimate partner, followed by an acquaintance or friend (9.2%); a child or other person known to victim, but the exact nature of the relationship was unclear (both 8.4%); or a parent (8.3%).

#### Precipitating Circumstances

Precipitating circumstances were identified in 69.1% of homicides (Table 5). One third of homicides with known circumstances were precipitated by an argument or conflict (33.9%), and 14.6% of homicides with known circumstances were related to intimate partner violence (Table 5). Intimate partner violence–related deaths include deaths related to conflict or violence between current or former intimate partners and also include deaths associated with intimate partner violence that are not deaths of the intimate partners themselves (e.g., a former boyfriend killing an ex-partner’s new boyfriend). Homicides also were commonly precipitated by another crime (22.9%); in 66.0% of those cases, the crime was in progress at the time of the incident. The most frequent types of precipitating crimes were assault or homicide (38.9%), robbery (32.9%), drug trade^§§^ (14.5%), burglary (10.8%), motor vehicle theft (5.0%), rape or sexual assault (2.3%), and arson (1.7%) (Supplementary Table S6, https://stacks.cdc.gov/view/cdc/127523). A physical fight between two persons (13.7%), a drive-by shooting (12.7%), and drug involvement (e.g., relating to a drug habit or illegal drug trafficking; 10.3%) were other common precipitating circumstances.

**TABLE 5:** Number* and percentage^†^ of homicides, by decedent sex and precipitating circumstances — National Violent Death Reporting System, 48 states^§^ and the District of Columbia, 2020

Precipitating circumstance	Male No. (%)	Female No. (%)	Total No. (%)
**Mental health and substance use**			
Other substance use problem (excludes alcohol)	1,499 (13.1)	300 (10.4)	**1,799 (12.6)**
Current diagnosed mental health problem	514 (4.5)	193 (6.7)	**707 (4.9)**
Alcohol problem	489 (4.3)	106 (3.7)	**595 (4.2)**
History of ever being treated for a mental health or substance use problem	272 (2.4)	102 (3.5)	**374 (2.6)**
Current mental health or substance use treatment	142 (1.2)	70 (2.4)	**212 (1.5)**
Other addiction (e.g., gambling or sexual)	72 (<1.0)	11 (<1.0)	**83 (<1.0)**
Current depressed mood	33 (<1.0)	18 (<1.0)	**51 (<1.0)**
**Interpersonal**			
Intimate partner violence related	898 (7.9)	1,190 (41.3)	**2,088 (14.6)**
Other relationship problem (nonintimate)	910 (8.0)	147 (5.1)	**1,057 (7.4)**
Family relationship problem	505 (4.4)	254 (8.8)	**759 (5.3)**
Jealousy (lovers’ triangle)	241 (2.1)	87 (3.0)	**328 (2.3)**
Victim of interpersonal violence during past month	125 (1.1)	156 (5.4)	**281 (2.0)**
Perpetrator of interpersonal violence during past month	201 (1.8)	14 (<1.0)	**215 (1.5)**
**Life stressor**			
Argument or conflict	3,961 (34.7)	876 (30.4)	**4,837 (33.9)**
Physical fight (two persons, not a brawl)	1,702 (14.9)	257 (8.9)	**1,959 (13.7)**
Crisis during previous or upcoming 2 weeks	523 (4.6)	220 (7.6)	**743 (5.2)**
History of child abuse or neglect	79 (<1.0)	49 (1.7)	**128 (<1.0)**
**Crime and criminal activity**			
Precipitated by another crime	2,722 (23.9)	542 (18.8)	**3,264 (22.9)**
Crime in progress^¶^	1,822 (66.9)	333 (61.4)	**2,155 (66.0)**
Drug involvement	1,301 (11.4)	173 (6.0)	**1,474 (10.3)**
Gang related	1,004 (8.8)	96 (3.3)	**1,100 (7.7)**
**Homicide event**			
Drive-by shooting	1,605 (14.1)	208 (7.2)	**1,813 (12.7)**
Walk-by assault	955 (8.4)	108 (3.8)	**1,063 (7.4)**
Victim used a weapon	963 (8.4)	39 (1.4)	**1,002 (7.0)**
Random violence	498 (4.4)	114 (4.0)	**612 (4.3)**
Caretaker abuse or neglect led to death	308 (2.7)	258 (9.0)	**566 (4.0)**
Justifiable self-defense	442 (3.9)	11 (<1.0)	**453 (3.2)**
Mentally ill suspect	192 (1.7)	181 (6.3)	**373 (2.6)**
Victim was a bystander	216 (1.9)	126 (4.4)	**342 (2.4)**
Brawl	272 (2.4)	12 (<1.0)	**284 (2.0)**
Victim was an intervener assisting a crime victim	147 (1.3)	23 (<1.0)	**170 (1.2)**
Stalking	22 (<1.0)	39 (1.4)	**61 (<1.0)**
Prostitution	25 (<1.0)	23 (<1.0)	**48 (<1.0)**
Victim was a police officer on duty	29 (<1.0)	3 (<1.0)	**32 (<1.0)**
Hate crime	14 (<1.0)	1 (<1.0)	**15 (<1.0)**
Mercy killing	1 (<1.0)	10 (<1.0)	**11 (<1.0)**
Total**	**11,405 (68.0)**	**2,879 (73.9)**	**14,284 (69.1)**

* Includes homicides with one or more precipitating circumstances. Total numbers do not equal the sums of the columns because more than one circumstance could have been present per decedent.

^†^ Denominator includes those homicides with one or more precipitating circumstances. The sums of percentages in columns exceed 100% because more than one circumstance could have been present per decedent.

^§^ Includes all U.S. states, with exception of Florida and Hawaii. Data for California are for violent deaths that occurred in 35 counties (Amador, Butte, Colusa, Contra Costa, Fresno, Glenn, Humboldt, Imperial, Kern, Kings, Lassen, Lake, Los Angeles, Marin, Mendocino, Merced, Modoc, Mono, Orange, Placer, Sacramento, San Benito, San Diego, San Francisco, San Mateo, Santa Cruz, Shasta, Siskiyou, Solano, Sonoma, Stanislaus, Tehama, Trinity, Ventura, and Yolo). Data for Texas are for violent deaths that occurred in four counties (Bexar, Dallas, Harris, and Tarrant).

^¶^ Denominator includes those decedents involved in an incident that was precipitated by another crime.

** Circumstances were unknown for 6,397 decedents (5,379 males and 1,018 females); total number of homicide decedents = 20,681 (16,784 males and 3,897 females).

Among the identified homicide circumstances, several differences were noted by decedent’s sex, and intimate partner violence accounted for the largest percentage difference. Intimate partner violence was a precipitating circumstance for approximately 41.3% of homicides among females but only 7.9% of homicides among males (Table 5). In incidents where intimate partner violence was a precipitating circumstance and victim-suspect relationship was known, the suspect was a current or former intimate partner in 92.8% of homicides among females and 50.3% of homicides among males. Females were more often the direct victims of intimate partner violence–related homicides, whereas males were more often corollary victims. A larger proportion of homicides of females than males also resulted from caregiver abuse or neglect (9.0% versus 2.7%) or were perpetrated by a suspect with a mental health problem (e.g., schizophrenia or other psychotic conditions, depression, or posttraumatic stress disorder) (6.3% versus 1.7%). A larger proportion of homicides of males than females were preceded by a physical fight (14.9% versus 8.9%), involved drugs (11.4% versus 6.0%), or were gang related (8.8% versus 3.3%). A larger proportion of male homicide victims (8.4%) than female homicide victims (1.4%) also were reported to have used a weapon during the incident.

### Legal Intervention Deaths

#### Sex, Age Group, and Race and Ethnicity

For 2020, a total of 48 NVDRS states (46 states collecting statewide data, 35 California counties, and four Texas counties) and the District of Columbia collected data on 868 incidents involving 874 legal intervention deaths (Supplementary Table S1, https://stacks.cdc.gov/view/cdc/127523). The highest rate of legal intervention death by age group was among men aged 30–34 years (1.5 per 100,000 population), followed by men aged 25–29 years (1.4 per 100,000 population) and 35–44 years (1.2 per 100,000 population) (Table 6). Approximately all legal intervention deaths were among males (96.2%). Although White males accounted for nearly one half (49.1%) of all legal intervention deaths, AI/AN males had the highest legal intervention death rate (3.1 per 100,000 population), representing a rate 6.2 times that of White males (0.5 per 100,000 population). The legal intervention death rate for Black males (1.2 per 100,000 population) was 2.4 times the rate for White males. The legal intervention death rate for Hispanic males was 0.7 per 100,000 population.

**TABLE 6:** Number, percentage,* and rate^†^ of legal intervention^§^ deaths, by selected demographic characteristics of decedent, method of injury used, and location in which injury occurred — National Violent Death Reporting System, 48 states^¶^ and the District of Columbia, 2020

Characteristic	Male		Female		Total	
	No. (%)	Rate	No. (%)	Rate	No. (%)	Rate
**Age group, yrs**						
<10	0 (0.0)	—**	0 (0.0)	—	**0 (0.0)**	**—**
10–14	1 (<1.0)	—	0 (0.0)	—	**1 (<1.0)**	**—**
15–19	40 (4.8)	0.4	3 (9.1)	—	**43 (4.9)**	**0.2**
20–24	78 (9.3)	0.8	2 (6.1)	—	**80 (9.2)**	**0.4**
25–29	136 (16.2)	1.4	6 (18.2)	—	**142 (16.2)**	**0.7**
30–34	144 (17.1)	1.5	7 (21.2)	—	**151 (17.3)**	**0.8**
35–44	215 (25.6)	1.2	9 (27.3)	—	**224 (25.6)**	**0.6**
45–54	132 (15.7)	0.8	2 (6.1)	—	**134 (15.3)**	**0.4**
55–64	71 (8.4)	0.4	3 (9.1)	—	**74 (8.5)**	**0.2**
65–74	18 (2.1)	—	1 (3.0)	—	**19 (2.2)**	**—**
75–84	6 (<1.0)	—	0 (0.0)	—	**6 (<1.0)**	**—**
≥85	0 (0.0)	—	0 (0.0)	—	**0 (0.0)**	**—**
**Race and ethnicity^††^**						
American Indian or Alaska Native	39 (4.6)	3.1	1 (3.0)	—	**40 (4.6)**	**1.6**
Asian or Pacific Islander	11 (1.3)	—	1 (3.0)	—	**12 (1.4)**	**—**
Black or African American	219 (26.0)	1.2	2 (6.1)	—	**221 (25.3)**	**0.6**
White	413 (49.1)	0.5	21 (63.6)	<0.1	**434 (49.7)**	**0.3**
Hispanic or Latino	156 (18.5)	0.7	8 (24.2)	—	**164 (18.8)**	**0.4**
Other	1 (<1.0)	—	0 (0.0)	—	**1 (<1.0)**	**—**
Unknown	2 (<1.0)	—	0 (0.0)	—	**2 (<1.0)**	**—**
**Method of injury**						
Firearm	724 (86.1)	0.5	21 (63.6)	<0.1	**745 (85.2)**	**0.3**
Motor vehicles (e.g., buses, motorcycles, or other transport vehicles)	41 (4.9)	<0.1	3 (9.1)	—	**44 (5.0)**	**<0.1**
Blunt instrument	11 (1.3)	—	4 (12.1)	—	**15 (1.7)**	**—**
Personal weapons (e.g., hands, feet, or fists)	7 (<1.0)	—	0 (0.0)	—	**7 (<1.0)**	**—**
Poisoning	4 (<1.0)	—	0 (0.0)	—	**4 (<1.0)**	**—**
Hanging, strangulation, or suffocation	4 (<1.0)	—	0 (0.0)	—	**4 (<1.0)**	**—**
Fall	3 (<1.0)	—	1 (3.0)	—	**4 (<1.0)**	**—**
Drowning	4 (<1.0)	—	0 (0.0)	—	**4 (<1.0)**	**—**
Fire or burns	1 (<1.0)	—	1 (3.0)	—	**2 (<1.0)**	**—**
Sharp instrument	1 (<1.0)	—	0 (0.0)	—	**1 (<1.0)**	**—**
Other (e.g., Taser, electrocution, or nail gun)	10 (1.2)	—	0 (0.0)	—	**10 (1.1)**	**—**
Unknown	31 (3.7)	—	3 (9.1)	—	**34 (3.9)**	**—**
**Location of injury**						
House or apartment	299 (35.6)	0.2	11 (33.3)	—	**310 (35.5)**	**0.1**
Street or highway	221 (26.3)	0.2	4 (12.1)	—	**225 (25.7)**	**<0.1**
Motor vehicle	76 (9.0)	<0.1	8 (24.2)	—	**84 (9.6)**	**<0.1**
Parking lot, public garage, or public transport	51 (6.1)	<0.1	1 (3.0)	—	**52 (5.9)**	**<0.1**
Commercial or retail area	33 (3.9)	<0.1	0 (0.0)	—	**33 (3.8)**	**<0.1**
Natural area	27 (3.2)	<0.1	0 (0.0)	—	**27 (3.1)**	**<0.1**
Hotel or motel	8 (<1.0)	—	1 (3.0)	—	**9 (1.0)**	**—**
Jail or prison	7 (<1.0)	—	1 (3.0)	—	**8 (<1.0)**	**—**
Other location^§§^	55 (6.5)	—	0 (0.0)	—	**55 (6.3)**	**—**
Unknown	64 (7.6)	—	7 (21.2)	—	**71 (8.1)**	**—**
**Total**	**841 (100)**	**0.6**	**33 (100)**	**<0.1**	**874 (100)**	**0.3**

* Percentages might not total 100% because of rounding.

^†^ Per 100,000 population.

^§^ The term legal intervention does not denote the lawfulness or legality of the circumstances surrounding the death.

^¶^ Includes all U.S. states, with exception of Florida and Hawaii. Data for California are for violent deaths that occurred in 35 counties (Amador, Butte, Colusa, Contra Costa, Fresno, Glenn, Humboldt, Imperial, Kern, Kings, Lassen, Lake, Los Angeles, Marin, Mendocino, Merced, Modoc, Mono, Orange, Placer, Sacramento, San Benito, San Diego, San Francisco, San Mateo, Santa Cruz, Shasta, Siskiyou, Solano, Sonoma, Stanislaus, Tehama, Trinity, Ventura, and Yolo). Data for Texas are for violent deaths that occurred in four counties (Bexar, Dallas, Harris, and Tarrant). Denominators for the rates for California and Texas represent only the populations of the counties from which the data were collected.

** Dashes indicate cell data are suppressed because number of decedents is <20 or characteristic response is “Other” or “Unknown.”

^††^ Persons of Hispanic or Latino (Hispanic) origin might be of any race but were categorized as Hispanic; all racial groups were non-Hispanic.

^§§^ Other location includes (in descending order): hospital or medical facility, park/playground/sports or athletic area, railroad tracks, industrial or construction area, office building, preschool/school/college/school bus, synagogue/church/temple, supervised residential facility, bar/nightclub, abandoned house/building/ warehouse, farm, bridge, cemetery/graveyard/other burial ground, and other unspecified location.

#### Method and Location of Injury

A firearm was used in a majority (85.2%) of legal intervention deaths (Table 6). Legal intervention deaths occurred most frequently in a house or apartment (35.5%), followed by a street or highway (25.7%) or a motor vehicle (9.6%).

#### Precipitating Circumstances

Precipitating circumstances were identified in 90.4% of legal intervention deaths (Table 7). The decedent reportedly used a weapon in 69.4% of legal intervention death cases. In 25.8% of legal intervention deaths with known circumstances, a substance use problem (other than alcohol) was reported as a contributing factor, and 20.1% of decedents reportedly had a current diagnosed mental health problem. An argument or conflict or physical fight precipitated 15.4% and 6.7% of legal intervention deaths, respectively. A recent or impending crisis during the previous or upcoming 2 weeks was reported in 7.6% of legal intervention deaths. Among legal intervention deaths with known circumstances, intimate partner violence (10.3%), being a perpetrator of interpersonal violence during the past month (12.7%), family relationship problems (5.6%), and drug involvement (4.6%) were other notable precipitating circumstances.

**TABLE 7:** Number* and percentage^†^ of legal intervention^§^ deaths, by decedent sex and precipitating circumstances — National Violent Death Reporting System, 48 states^¶^ and the District of Columbia, 2020

Precipitating circumstance	Male	Female	Total
	No. (%)	No. (%)	No. (%)
**Mental health and substance use**			
Other substance use problem (excludes alcohol)	194 (25.3)	10 (41.7)	**204 (25.8)**
Current diagnosed mental health problem	151 (19.7)	8 (33.3)	**159 (20.1)**
History of ever being treated for a mental health or substance use problem	103 (13.4)	5 (20.8)	**108 (13.7)**
Alcohol problem	94 (12.3)	3 (12.5)	**97 (12.3)**
Current mental health or substance use treatment	60 (7.8)	4 (16.7)	**64 (8.1)**
Current depressed mood	23 (3.0)	0 (—)	**23 (2.9)**
Other addiction (e.g., gambling or sexual)	11 (1.4)	0 (—)	**11 (1.4)**
**Interpersonal**			
Perpetrator of interpersonal violence during past month	99 (12.9)	1 (4.2)	**100 (12.7)**
Intimate partner violence related	79 (10.3)	2 (8.3)	**81 (10.3)**
Family relationship problem	40 (5.2)	4 (16.7)	**44 (5.6)**
Other relationship problem (nonintimate)	25 (3.3)	1 (4.2)	**26 (3.3)**
Victim of interpersonal violence during past month	2 (<1.0)	1 (4.2)	**3 (<1.0)**
Jealousy (lovers’ triangle)	1 (<1.0)	0 (0.0)	**1 (<1.0)**
**Life stressor**			
Argument or conflict	119 (15.5)	3 (12.5)	**122 (15.4)**
Crisis during previous or upcoming 2 weeks	58 (7.6)	2 (8.3)	**60 (7.6)**
Physical fight (two persons, not a brawl)	53 (6.9)	0 (0.0)	**53 (6.7)**
History of child abuse or neglect	4 (<1.0)	0 (0.0)	**4 (<1.0)**
**Crime and criminal activity**			
Drug involvement	34 (4.4)	2 (8.3)	**36 (4.6)**
Gang related	11 (1.4)	0 (0.0)	**11 (1.4)**
**Homicide event**			
Victim used a weapon	533 (69.6)	15 (62.5)	**548 (69.4)**
Random violence	6 (<1.0)	0 (0.0)	**6 (<1.0)**
Brawl	4 (<1.0)	0 (0.0)	**4 (<1.0)**
Stalking	4 (<1.0)	0 (0.0)	**4 (<1.0)**
Walk-by assault	3 (<1.0)	0 (0.0)	**3 (<1.0)**
Drive-by shooting	2 (<1.0)	0 (0.0)	**2 (<1.0)**
Mentally ill suspect	1 (<1.0)	0 (0.0)	**1 (<1.0)**
Total**	**766 (91.1)**	**24 (72.7)**	**790 (90.4)**

* Includes deaths with one or more precipitating circumstances. Total numbers do not equal the sums of the columns because more than one circumstance could have been present per decedent.

^†^ Denominator includes those deaths with one or more precipitating circumstances. The sums of percentages in columns exceed 100% because more than one circumstance could have been present per decedent.

^§^ The term legal intervention does not denote the lawfulness or legality of the circumstances surrounding the death.

^¶^ Includes all U.S. states, with exception of Florida and Hawaii. Data for California are for violent deaths that occurred in 35 counties (Amador, Butte, Colusa, Contra Costa, Fresno, Glenn, Humboldt, Imperial, Kern, Kings, Lassen, Lake, Los Angeles, Marin, Mendocino, Merced, Modoc, Mono, Orange, Placer, Sacramento, San Benito, San Diego, San Francisco, San Mateo, Santa Cruz, Shasta, Siskiyou, Solano, Sonoma, Stanislaus, Tehama, Trinity, Ventura, and Yolo). Data for Texas are for violent deaths that occurred in four counties (Bexar, Dallas, Harris, and Tarrant).

** Circumstances were unknown for 84 decedents (75 males and nine females); total number of legal intervention deaths = 874 (841 males and 33 females).

### Unintentional Firearm Deaths

#### Sex, Age Group, and Race and Ethnicity

In 2020, a total of 48 NVDRS states (46 states collecting statewide data, 35 California counties, and four Texas counties) and the District of Columbia collected data on 500 incidents involving 504 unintentional firearm deaths (Supplementary Table S1, https://stacks.cdc.gov/view/cdc/127523). Nearly one half (n = 225; 44.6%; data not shown) of these deaths were self-inflicted, and 170 deaths (33.7%; data not shown) were known to be inflicted by another person; for the remaining 109 deaths (21.6%; data not shown), whether the injury was inflicted by the decedent or by another person was unknown. Males accounted for 86.1% of decedents (Table 8). Persons aged ≤24 years accounted for more than one half (55.4%) of all unintentional firearm deaths. The majority of decedents were White persons (52.8%), followed by Black persons (33.1%).

**TABLE 8:** Number and percentage* of unintentional firearm deaths, by selected demographic characteristics of decedent, location of injury, and type of firearm — National Violent Death Reporting System, 48 states^†^ and the District of Columbia, 2020

Characteristic	No. (%)
**Sex**	
Male	434 (86.1)
Female	70 (13.9)
**Race and ethnicity^§^**	
American Indian or Alaska Native	12 (2.4)
Asian or Pacific Islander	6 (1.2)
Black or African American	167 (33.1)
White	266 (52.8)
Other	2 (<1.0)
Hispanic or Latino	51 (10.1)
**Age group, yrs**	
<1	0 (—)
1–4	49 (9.7)
5–9	26 (5.2)
10–14	47 (9.3)
15–19	85 (16.9)
20–24	72 (14.3)
25–29	40 (7.9)
30–34	30 (6.0)
35–44	42 (8.3)
45–54	38 (7.5)
55–64	32 (6.3)
65–74	28 (5.6)
75–84	12 (2.4)
≥85	3 (<1.0)
**Location of injury**	
House or apartment	378 (75.0)
Motor vehicle	29 (5.8)
Natural area	23 (4.6)
Street or highway	9 (1.8)
Hotel or motel	8 (1.6)
Commercial or retail area	7 (1.4)
Other location^¶^	20 (4.0)
Unknown	30 (6.0)
**Firearm type**	
Handgun	302 (59.9)
Rifle	44 (8.7)
Shotgun	34 (6.7)
Other firearm type	0 (—)
Unknown	124 (24.6)
**Total**	**504 (100)**

* Percentages might not total 100% because of rounding.

^†^ Includes all U.S. states, with exception of Florida and Hawaii. Data for California are for violent deaths that occurred in 35 counties (Amador, Butte, Colusa, Contra Costa, Fresno, Glenn, Humboldt, Imperial, Kern, Kings, Lassen, Lake, Los Angeles, Marin, Mendocino, Merced, Modoc, Mono, Orange, Placer, Sacramento, San Benito, San Diego, San Francisco, San Mateo, Santa Cruz, Shasta, Siskiyou, Solano, Sonoma, Stanislaus, Tehama, Trinity, Ventura, and Yolo). Data for Texas are for violent deaths that occurred in four counties (Bexar, Dallas, Harris, and Tarrant).

^§^ Persons of Hispanic or Latino (Hispanic) origin might be of any race but were categorized as Hispanic; all racial groups were non-Hispanic.

^¶^ Other location includes (in descending order): parking lot/public garage/public transport, park/playground/sports or athletic area, farm, bar/nightclub, industrial or construction area, preschool/school/college/school bus, supervised residential facility, and other unspecified location.

#### Location of Injury and Firearm Type

Among unintentional firearm deaths, 75.0% occurred in a house or apartment, followed by a motor vehicle (5.8%) or a natural area (4.6%) (Table 8). The majority of unintentional firearm deaths involved a handgun (59.9%), followed by a rifle (8.7%) or a shotgun (6.7%). The firearm type was unknown in approximately one quarter (24.6%) of unintentional firearm deaths.

#### Context and Circumstances of Injury

The context and circumstances of injury were identified in 81.3% of unintentional firearm deaths (Table 9). Among those with context and circumstance information, the context of injury for nearly one half (47.1%) of unintentional firearm deaths was playing with a gun. Other contexts of injury were showing the gun to others (11.2%), cleaning the gun (7.3%), and loading or unloading the gun (4.6%). Approximately one fourth (27.8%) of unintentional firearm deaths were caused by a person unintentionally pulling the trigger; 10.5% were caused by a person mistakenly thinking the gun was unloaded, and 8.0% of deaths were because of the gun being mistaken for a toy.

**TABLE 9:** Number and percentage* of unintentional firearm deaths, by context and circumstances of injury — National Violent Death Reporting System, 48 states^†^ and the District of Columbia, 2020

Characteristic	No. (%)
**Context of injury**	
Playing with gun	193 (47.1)
Showing gun to others	46 (11.2)
Cleaning gun	30 (7.3)
Loading or unloading gun	19 (4.6)
Hunting	17 (4.1)
Target shooting	7 (1.7)
Celebratory firing	1 (<1.0)
Other context of injury	100 (24.4)
**Circumstance of injury**	
Unintentionally pulled trigger	114 (27.8)
Thought gun was unloaded	43 (10.5)
Gun was mistaken for a toy	33 (8.0)
Thought unloaded, magazine disengaged	22 (5.4)
Gun was dropped	19 (4.6)
Thought gun safety was engaged	11 (2.7)
Gun fired while holstering	10 (2.4)
Gun fired because of defect or malfunction	7 (1.7)
Bullet ricocheted	2 (<1.0)
Gun fired while handling safety lock	2 (<1.0)
Other mechanism of injury	65 (15.9)
**Total^§^**	**410 (81.3)**

* Percentages might exceed 100% because one or more circumstances could have been known per death. Number and percentage are reported when the number of deaths is <5 because no particular circumstance identifies a single death. Denominator includes those deaths with one or more precipitating circumstances.

^†^ Includes all U.S. states, with exception of Florida and Hawaii. Data for California are for violent deaths that occurred in 35 counties (Amador, Butte, Colusa, Contra Costa, Fresno, Glenn, Humboldt, Imperial, Kern, Kings, Lassen, Lake, Los Angeles, Marin, Mendocino, Merced, Modoc, Mono, Orange, Placer, Sacramento, San Benito, San Diego, San Francisco, San Mateo, Santa Cruz, Shasta, Siskiyou, Solano, Sonoma, Stanislaus, Tehama, Trinity, Ventura, and Yolo). Data for Texas are for violent deaths that occurred in four counties (Bexar, Dallas, Harris, and Tarrant).

^§^ Circumstances were unknown for 94 decedents; total number of unintentional firearm decedents = 504.

### Deaths of Undetermined Intent

#### Sex, Age Group, and Race and Ethnicity

In 2020, a total of 48 NVDRS states (46 states collecting statewide data, 35 California counties, and four Texas counties) and the District of Columbia collected data on 5,386 incidents involving 5,429 deaths of undetermined intent (Supplementary Table S1, https://stacks.cdc.gov/view/cdc/127523). The overall rate of deaths of undetermined intent was 2.0 per 100,000 population (Supplementary Table S10, https://stacks.cdc.gov/view/cdc/127523). The rate of deaths of undetermined intent was higher among males (2.7 per 100,000 population) than among females (1.2 per 100,000 population). Approximately two thirds (69.8%) of deaths of undetermined intent were among adults aged 30–64 years. The rate of deaths of undetermined intent was highest among males aged 30–34 years (4.7 per 100,000 population), followed by males aged 35–44 and 45–54 years (both 4.3 per 100,000 population) and 55–64 years (3.9 per 100,000 population). The rate of deaths of undetermined intent among infants (i.e., children aged <1 year) was 2.7 per 100,000 population. Although White persons accounted for the majority (63.7%; 2.0 per 100,000 population) of deaths of undetermined intent, AI/AN persons had the highest rate (4.3 per 100,000 population). Among males, AI/AN males (5.6 per 100,000 population) and Black males (5.4 per 100,000 population) had the highest rate of deaths of undetermined intent. Among females, AI/AN females also had the highest rate of deaths of undetermined intent (3.1 per 100,000 population), followed by Black females (1.7 per 100,000 population).

#### Method and Location of Injury

Poisoning was the most common method of injury in deaths of undetermined intent (66.4%), followed by firearm (4.5%); drowning (4.1%); blunt instrument (2.9%); fall (2.8%); motor vehicle (2.7%); and fire or burns or hanging, strangulation, or suffocation (2.0% each). Personal weapons, sharp instruments, intentional neglect, shaking, and other methods were each used as method of injury in <1.0% of undetermined intent deaths (Supplementary Table S10, https://stacks.cdc.gov/view/cdc/127523). Weapon type was unknown for 10.0% of undetermined intent deaths. The majority of deaths of undetermined intent occurred in a house or apartment (63.8%), followed by a street or highway (4.8%), a natural area (4.7%), or a hotel or motel (4.0%).

#### Toxicology Results of Decedent

Toxicology tests for BAC were conducted for 70.7% of decedents in deaths of undetermined intent (Supplementary Table S11, https://stacks.cdc.gov/view/cdc/127523). Among those with positive results for alcohol (37.4%), 47.2% had a BAC ≥0.08 g/dL. Tests for the following substances were conducted for the percentage of decedents indicated in parentheses: amphetamines (36.5%), antidepressants (34.3%), benzodiazepines (38.3%), cannabis (commonly referred to as marijuana; 33.0%), cocaine (46.4%), and opioids (71.0%). Among decedents tested for amphetamines, 34.3% had positive test results. Among those tested for antidepressants, 52.1% had positive results at the time of death; 40.8% of those tested for benzodiazepines had positive results, 34.5% of those tested for cannabis had positive results, and 43.4% of those tested for cocaine had positive results. Results for opioids (illicit or prescription) were positive in 76.9% of decedents tested. Carbon monoxide was tested for a substantially smaller proportion of decedents (4.1%) but was identified in 65.3% of those decedents.

#### Precipitating Circumstances

Circumstances were identified in 78.0% of deaths of undetermined intent (Supplementary Table S12, https://stacks.cdc.gov/view/cdc/127523). Among deaths of undetermined intent with known circumstances, 34.3% of decedents had a current diagnosed mental health problem at time of death; depression or dysthymia (54.1%), anxiety disorder (27.2%), and bipolar disorder (21.0%) were the most common diagnoses among these decedents, and 7.9% had a current depressed mood. Substance use problems (other than alcohol; 68.8%) and alcohol problems (25.3%) were the most commonly reported circumstances. Among all deaths of undetermined intent, 20.3% were receiving mental health or substance use treatment at the time of death; 27.7% of decedents had a history of ever being treated for a mental health or substance use problem. Physical health problems (12.7%) and a recent or impending crisis during the preceding or upcoming 2 weeks (9.9%) were other life stressors identified in deaths of undetermined intent (Supplementary Table S13, https://stacks.cdc.gov/view/cdc/127523). Among decedents, 10.5% had a history of suicidal thoughts or plans, 7.3% had a history of attempting suicide, and 4.9% had disclosed intent to die by suicide.

### Violent Deaths in Puerto Rico

For 2020, Puerto Rico collected data on 729 incidents involving 790 deaths (data not shown). Homicide (n = 550) accounted for the largest proportion (69.6%) and highest rate (16.8 per 100,000 population) of violent deaths, followed by suicide (n = 210; 26.6%; 7.0 per 100,000 population aged ≥10 years) (Supplementary Tables S14 and S17, https://stacks.cdc.gov/view/cdc/127523).

### Homicides

#### Sex, Age Group, and Race and Ethnicity

In 2020, a total of 500 homicides among males and 49 homicides among females were reported in Puerto Rico (Supplementary Table S14, https://stacks.cdc.gov/view/cdc/127523). The overall homicide rate for males (32.2 per 100,000 population) was 11.5 times the rate for females (2.8 per 100,000 population). Among males, the homicide rate was 79.8 per 100,000 population among adults aged 18–29 years and 66.5 per 100,000 population among those aged 30–44 years. Most (94.5%) homicide victims were Hispanic.

#### Method, Location of Injury, and Victim-Suspect Relationship

A firearm was used in a majority (88.9%) of homicides (Supplementary Table S14, https://stacks.cdc.gov/view/cdc/127523). A firearm was the most common method used in homicides of both males (89.6%) and females (83.7%); however, the firearm homicide rate for males (28.8 per 100,000 population) was 12 times the rate for females (2.4 per 100,000 population). Among males, a street or highway was the most common location (49.0%) of homicides, whereas a house or apartment was the most common location (40.8%) of homicides for females.

The victim-suspect relationship was known for 48.9% of homicides (Supplementary Table S14, https://stacks.cdc.gov/view/cdc/127523). When the relationship was known, the suspect for male victims was most often a person known to the victim, but the exact nature of the relationship was unclear (39.5%), followed by a rival gang member (30.5%). Among females, the suspect was most often a current or former intimate partner (38.9%), followed by a person known to the victim, but the exact nature of the relationship was unclear (27.8%).

#### Toxicology Results of Decedent

Tests for BAC were conducted for 99.3% of homicide decedents (Supplementary Table S15, https://stacks.cdc.gov/view/cdc/127523). Among those with positive results for alcohol (26.6%), 34.5% had a BAC ≥0.08 g/dL. Tests for cocaine, cannabis (commonly referred to as marijuana), and opioids were conducted for 99.3%, 75.1%, and 99.3% of decedents, respectively. Results for cocaine, cannabis, and opioids were positive in 18.1%, 31.0%, and 9.0% of decedents tested, respectively.

#### Precipitating Circumstances

Precipitating circumstances were identified in 97.3% of homicides (Supplementary Table S16, https://stacks.cdc.gov/view/cdc/127523). Among males, more than one half (50.3%) of homicides were gang related, 43.1% involved drugs, and approximately one fourth (22.0%) involved drive-by shootings. Intimate partner violence was identified as a contributing factor in 9.0% of homicides overall; approximately one third (33.3%) of homicides among females were precipitated by intimate partner violence, compared with 6.6% of homicides among males.

### Suicides

#### Sex, Age Group, and Race and Ethnicity

In 2020, a total of 210 suicides among persons aged ≥10 years (178 suicides among males and 32 suicides among females) were reported in Puerto Rico (Supplementary Table S17, https://stacks.cdc.gov/view/cdc/127523). The suicide rate for males was 6.2 times the rate for females (12.5 versus 2.0 per 100,000 population aged ≥10 years). The suicide rates were highest among men aged ≥65 years (16.3 per 100,000 population aged ≥10 years), 45–64 years (16.2 per 100,000 population aged ≥10 years), and 30–44 years (13.5 per 100,000 population aged ≥10 years). The majority (90.5%) of suicide decedents overall were Hispanic.

#### Method and Location of Injury

Hanging, strangulation, or suffocation was the most commonly used method for suicide among both males (66.9%) and females (50.0%) (Supplementary Table S17, https://stacks.cdc.gov/view/cdc/127523). A firearm was used in 18.0% of suicides among males. The most common location where a suicide took place was a house or apartment both for males (76.4%) and females (93.8%).

#### Toxicology Results of Decedent

Tests for BAC were conducted for 97.6% of suicide decedents (Supplementary Table S18, https://stacks.cdc.gov/view/cdc/127523). Among those with positive results for alcohol (29.3%), 60.0% had a BAC ≥0.08 g/dL. Other than alcohol, suicide decedents were most often tested for cocaine (97.1%), cannabis (commonly referred to as marijuana; 65.2%), and opioids (97.1%). Results for cocaine, cannabis, and opioids were positive in 12.3%, 8.8%, and 6.4% of decedents tested, respectively.

#### Precipitating Circumstances

Circumstances were identified in 93.8% of suicides (Supplementary Table S19, https://stacks.cdc.gov/view/cdc/127523). Overall, a mental health problem was the most common circumstance among suicide decedents, with 52.8% having a current diagnosed mental health problem and 49.7% experiencing a depressed mood at the time of death.

Among males, 49.7% of suicide decedents had a current depressed mood, and 49.1% had a current diagnosed mental health problem. Depression or dysthymia was most often the mental health diagnosis experienced by male suicide decedents with a diagnosed mental health problem (77.8%), followed by anxiety disorder (18.5%). Approximately one third (31.5%) of male suicide decedents had a history of ever being treated for a mental health or substance use problem. Approximately one fourth (23.0%) of male suicide decedents had a history of expressing suicidal thoughts and plans, and 22.4% had a history of attempting suicide. Other precipitating circumstances for male suicide decedents included physical health problem (17.6%) and intimate partner problems (15.2%).

Among female suicide decedents, 50.0% had a current depressed mood, and 71.9% had a current diagnosed mental health problem. Depression or dysthymia was most often the mental health diagnosis experienced by female suicide decedents who had a diagnosed mental health problem (73.9%). One half (50.0%) of female decedents had a history of ever being treated for a mental health or substance use problem, 40.6% were known to have been receiving mental health or substance use treatment at the time of death, and 34.4% had a history of attempting suicide.

## Discussion

Violent deaths affect all subgroups, regardless of sex, age, or race and ethnicity. NVDRS provides information on specific manners of violent death and can be used to describe characteristics of inequities experienced by populations particularly affected by fatal violence. NVDRS data also can identify common risk factors for multiple forms of violence. These details increase the knowledge base about the circumstances associated with violence and can assist public health authorities and their partners in developing and guiding effective, data-driven approaches to violence prevention.

The occurrence of violent death varies greatly across states, the District of Columbia, and Puerto Rico (*1*). This report summarizes data on violent deaths that occurred in 2020 in 48 NVDRS states, the District of Columbia, and Puerto Rico and describes selected characteristics. The 48 states and the District of Columbia represented 84.0% of the U.S. population (*9*) and accounted for 84.7% of violent deaths in the United States in 2020 (*1*). NVDRS contributes to the national prevention initiative Healthy People 2020 objectives to increase the number of states that link data on violent deaths from death certificates, coroner or medical examiner records, and law enforcement reports at state and local levels and the Healthy People 2030 objectives to reduce the number of suicides, homicides, and firearm-related deaths (*14*,*15*).

Violence is preventable and reducing violent deaths in communities is possible with evidence-based approaches (*16*). CDC developed resources for action (i.e., technical packages) to assist communities in identifying violence prevention approaches that are based on the best available evidence. The resources for action describe strategies and specific programs, practices, and policies with evidence to reduce the risk for suicide, youth violence, child abuse and neglect, adverse childhood experiences, intimate partner violence, and sexual violence (*17*–*22*). Each resource for action considers the multifaceted and interactive effects of the different levels of social-ecological interrelationships, including individual, relationship, family, school, and community factors that influence violence-related outcomes. NVDRS gathers ongoing, systematic, and consistent data on violent deaths that can be used by violence prevention experts within their communities to guide planning and implementation and track outcomes of violence prevention strategies and approaches.

### Suicides

#### Suicide Circumstances

Approximately one third of suicide decedents had a history of suicidal thoughts or plans, and approximately one fourth had disclosed their suicidal intent. Multiple factors contribute to the risk for suicide (*23*), and the findings in this report indicate that intimate partner problems, recent or impending crises, arguments or conflicts, and physical and mental health problems were common precipitating circumstances. The most commonly identified circumstance was mental health problems, yet approximately one half of suicide decedents did not have a known diagnosed mental health condition at the time of death. Past suicidal behavior and mental health problems are well documented as important risk factors to emphasize in suicide prevention (*19*,*24*). Less than one fourth of suicide decedents were known to be receiving treatment at the time of death, indicating a gap between those receiving treatment and those who would likely benefit from it.

Mental health problems and substance use also often co-occur among suicide victims (*25*,*26*). In this analysis, alcohol use, especially alcohol use in excess of states’ legal limit (BAC>0.08 g/dL), was frequently observed among suicide decedents who were tested for substances. Alcohol use is a strong predictor of suicidal behavior (*27*), victimization (*28*), and interpersonal violence perpetration (*29*). Intoxication can cause disinhibition, enhanced feelings of hopelessness and depression, and impaired judgment, which can lead to impulsive behaviors (*24*). In addition, positive toxicology results for opioids (illicit or prescription) were reported in approximately one fourth of suicide decedents tested for these substances. In 2017, opioid overdose was recognized as a public health emergency (*30*) after increases in opioid overdose deaths (*31*). As a result, CDC has implemented comprehensive surveillance and prevention activities through the Overdose Data to Action cooperative agreement to support state, territorial, county, and city health departments in collecting and reporting more timely and complete data on overdose morbidity and mortality and using the data to guide prevention and response efforts (*32*). In addition, the Opioid Rapid Response Program was introduced to facilitate care coordination, risk reduction, and other overdose prevention activities across federal and state health agencies to mitigate overdose risks among patients who lose access to opioid prescribers or medications for opioid use disorder (*33*). Previous research also has suggested that chronic pain might be a contributor to suicide (*34*). The CDC Clinical Practice Guideline for Prescribing Opioids for Pain (*35*), updated in 2022, is a clinical tool to help clinicians and patients work together to make informed, patient-centered decisions about pain care. The guideline is intended to improve communication between clinicians and patients about the benefits and risks of pain treatments (e.g., opioid therapy for pain), improve the safety and effectiveness of pain treatment, mitigate pain, improve function and quality of life for patients with pain, and reduce the risks associated with opioid pain therapy (e.g., opioid use disorder, overdose, and death). Other important activities to address the opioid overdose epidemic include expanding naloxone availability and access to treatment with medications for opioid use disorder, addressing prescription opioid misuse, enhancing public health and public safety partnerships, and maximizing the ability of health systems to link persons who use drugs to treatment and harm-reduction services (*32*,*34*,*36*–*38*).

Another factor that might contribute to the risk for suicide is access to lethal means (e.g., firearms) among persons at risk for suicide (*19*). A firearm was the most common method used in suicides, accounting for approximately one half of the deaths by suicide in this analysis. Lethal means provide limited opportunity for intervention and have high case-fatality rates (*19*). Males and older adults have been found to be more likely than females and younger adults, respectively, to use firearms as a means of suicide (*39*,*40*). This analysis found that suicide rates were highest among males and adults aged ≥75 years. Creating protective environments by reducing access to lethal means among persons at risk can be an effective strategy to prevent suicide (*19*).

#### Racial and Ethnic Inequities in Suicide Rates

Demographic variations persist in the manner of death from violence-related injuries. Suicides comprised the majority of violent deaths collected in NVDRS and occurred at higher rates among AI/AN and White persons compared with other racial and ethnic groups. The findings in this report regarding suicide rates experienced by AI/AN persons, in particular, warrant attention to the contextual factors that might contribute to higher rates of suicide, such as barriers to accessing mental health care, exposure to the suicide of a friend or family member as a contributing factor to a person’s own death by suicide, and alcohol and substance use (*41*). Among AI/AN persons, experiences with historical trauma related to the intergenerational, collective, and cumulative effect of colonialism and ongoing inequities (e.g., discrimination, disparaging stereotypes, and microaggressions) can contribute to risk for suicide (*42*,*43*). Challenges related to suicide, alcohol, and substance use are not inherent to AI/AN culture but can be interpreted within the context of historical racism and ongoing inequities. Furthermore, acknowledging the heterogeneity among persons and groups who identify as AI/AN is important (*41*,*42*).

#### The COVID-19 Pandemic and Suicide

Although studies using NVDRS data to examine the extent to which the COVID-19 pandemic has affected circumstances precipitating suicide are still ongoing, certain studies have highlighted the effect that the COVID-19 pandemic and mitigation measures (e.g., stay-at-home orders) have had on mental health, substance use, and suicide (*44*,*45*). With the experience of pandemic-related risk factors (e.g., social isolation, loss of income, and increased stress related to caregiver workload), U.S. adults reported elevated levels of anxiety disorder, depressive disorder, new or increased substance use, and suicidal ideation (*44*). Although one study found no substantial changes in age-adjusted firearm suicide rates among persons aged ≥10 years overall from 2019 to 2020, notable increases occurred in the firearm suicide rate among AI/AN persons and, specifically, AI/AN males aged 10–44 years (*45*). The impact of the COVID-19 pandemic underscored the need for increased access to health services promoting social connectedness and improved diagnostic and treatment resources for mental health and substance use problems (e.g., telehealth and harm reduction services) to mitigate increases in suicide ideation (*44*).

#### Suicide Prevention Strategies

Participating NVDRS states and jurisdictions have used VDRS data to generate reports and data visualizations to examine violent deaths and develop prevention efforts. For example, Oregon, Colorado, and Kentucky have used their VDRS data to guide suicide prevention efforts and generate reports highlighting where additional focus is needed. As part of the state’s campaign for gun safety and firearm-related death and injury prevention, Oregon VDRS used their data to develop a publicly available data dashboard to provide suicide and homicide trends and rates. The dashboard also highlights the state- and county-level implications of firearm mortality and presents these in conjunction with firearm safety tips and resources for clinicians (*46*). Using data from 2004–2020, Colorado published a report on suicide deaths and surrounding circumstances among first responders (e.g., firefighters, emergency medical services, and law enforcement) and last responders (e.g., coroners, death investigators, medical examiners, and morticians) (*47*). Colorado VDRS indicated that first and last responders were less likely to have been treated or have a diagnosed mental health condition compared with all Colorado suicide deaths, apart from posttraumatic stress disorder. Because of the unique nature of the work of first and last responders and their frequent exposure to trauma, the mental health needs of first and last responders must be better understood so suicide prevention efforts can be better tailored for these professions.

In addition, Kentucky VDRS published a report on their development of a novel theoretical framework that predicted suicide deaths during the COVID-19 pandemic starting from spring 2020 (*48*). In the report, Kentucky VDRS indicated that psychological and social effects of the COVID-19 pandemic (e.g., containment efforts and economic consequences) would negatively affect and create the perfect storm of conditions for suicide, especially in the most vulnerable populations, such as elderly persons, persons experiencing poverty, and persons with existing mental health problems. While the impacts of the COVID-19 pandemic are ongoing and continue to be assessed, the Kentucky VDRS program emphasized the importance of monitoring suicide trends locally and nationally to be proactive in developing suicide prevention efforts among populations most vulnerable to the psychological and social effects of the COVID-19 pandemic (*48*). CDC’s Suicide Prevention Resource for Action: A Compilation of the Best Available Evidence identifies the following seven strategies for reducing suicide and suicidal behaviors: 1) strengthen economic supports, 2) create protective environments, 3) improve access and delivery of suicide care, 4) promote healthy connections, 5) teach coping and problem-solving skills, 6) identify and support persons at risk, and 7) lessen harms and prevent future risk (*19*). These strategies support the goals and objectives of the National Strategy for Suicide Prevention (NSSP), a comprehensive national agenda for suicide prevention (*49*), and the National Action Alliance for Suicide Prevention’s priority to strengthen community-based prevention (*50*). NVDRS is relevant to the NSSP goals of increasing timeliness and usefulness of surveillance systems related to suicide prevention and evaluating outcomes and effectiveness of suicide prevention interventions. The suicide prevention resource for action includes examples of specific approaches that communities can implement to use each strategy. The findings in this report underscore the importance of approaches outlined in the resource for action, such as social-emotional learning programs, enhanced parenting skills and family relationships, treatment for persons at risk for suicide, and treatment to prevent reattempts.

### Homicides

#### Homicides of Infants and Children

Although homicide rates for children varied across age, infants (i.e., children aged <1 year) experienced a higher homicide rate compared with children aged 1–14 years. Certain studies have found the highest risk for newborn and infant homicide is on the day of birth (*51*,*52*). Risk starts in infancy and continues throughout childhood, highlighting the need to prioritize strategies focused on the prevention and intervention of child abuse and neglect to reduce risk for morbidity and mortality (*21*). Child abuse and neglect often are associated with immediate physical injuries, emotional and psychological problems, involvement in risky health behaviors later in life, and a wide range of broader physical health challenges and long-term health consequences (*21*).

CDC’s Preventing Child Abuse and Neglect: A Technical Package for Policy, Norm, and Programmatic Activities identified the following evidence-based strategies and approaches: 1) strengthening economic supports for families, 2) changing social norms to support parents and positive parenting, 3) providing quality care and education early in life, 4) enhancing parenting skills to promote healthy child development, and 5) intervening to decrease harms and prevent future risk (*21*). Child abuse and neglect are preventable, and the specific approaches described in the technical package can help create safe, stable, and nurturing relationships and environments (*53*) to prevent physical, mental, and emotional injuries as well as homicides of infants and children. The lack of safe, stable, and nurturing relationships and environments, which are essential for promoting children’s health and well-being, puts children at risk for adverse childhood experiences including violence, abuse, or death.

CDC’s Preventing Adverse Childhood Experiences: Leveraging the Best Available Evidence is a comprehensive approach to preventing and mitigating the harms of adverse childhood experiences (*18*). Immediate and long-term harm of adverse childhood experiences can be lessened using multiple strategies, such as strengthening economic supports for families through work policies; promoting social norms that protect against violence and adversity via public education campaigns; ensuring a strong start for children through programs such as early childhood home visitation; quality and affordable child care, and preschool enrichment programs; connecting youths to caring adults and activities; and intervening with enhanced primary care or victim-centered services (*18*).

#### Racial and Ethnic Inequities in Homicide Rates

Racial and ethnic minority groups experience inequitable rates of violent injury and homicide, particularly among youths and young adult males (*54*). In the United States, among both males and females, AI/AN and Black persons experienced the highest rates of homicide. In Puerto Rico, the homicide rate was more than double the suicide rate, and male victims, who were predominantly Hispanic (94.6%), experienced homicide rates similar to and exceeding the homicide rates experienced by AI/AN and Hispanic males in the U.S. states and the District of Columbia. Racial and ethnic inequities in exposure to violence are pervasive and persistent and the elimination of these inequities should be prioritized (*54*). Racial and ethnic minority groups are disproportionately exposed to systemic inequities such as residential segregation, concentrated disadvantage, stress from experiencing racism, limited access to the best educational and employment opportunities, and other conditions that increase the risk for experiencing violence (*55*,*56*). For example, homicide rates for males in Puerto Rico have been attributed, in part, to living in communities that have been marginalized and the socioeconomic incentives of being involved in illegal means of income that are associated with high risks for violence (*57*).

Racial and ethnic minority youths often live in communities with concentrated poverty, stressed economies, residential instability, neighborhood disorganization, low community cohesion, and informal controls (*55*,*56*,*58*). All these conditions are associated with violence and violence-related injuries, and addressing the contextual factors at the structural, societal, and community levels that serve as risk factors can have broad and sustained effects in reducing racial and ethnic disparities in violence exposure (*3*,*20*,*55*,*56*,*58*). Disparity reduction strategies include policies and programs that strengthen economic and household stability (*20*,*59*), improve physical and social environments (*20*,*60*), and reduce the continuation of violence (*20*,*56*).

#### Intimate Partner Violence–Related Homicides

Homicides among males were most often precipitated by an argument or conflict or occurred during the enactment of a crime (predominately assault or homicide). In contrast, 41.3% of homicides among females were intimate partner violence related, and a current or former spouse or intimate partner was the most-commonly identified suspect for female homicide victims with known suspects. Estimates from the 2015 National Intimate Partner and Sexual Violence Survey indicated that approximately 80 million persons in the United States have experienced intimate partner violence in their lifetime; furthermore, one in four females in the United States experienced intimate partner violence (e.g., contact sexual violence, physical violence, or stalking by an intimate partner) and associated adverse impacts, including experience of fear or concern for safety, at some point in their lives (*61*). Intimate partner violence–related homicides warrant further research to determine the contextual factors and characteristics of these fatal incidents and how these contextual factors might vary by various demographic characteristics.

CDC’s intimate partner violence prevention resource for action, Preventing Intimate Partner Violence Across the Lifespan: A Technical Package of Programs, Policies, and Practices, outlines multiple strategies for programs and policies to prevent intimate partner violence and to decrease harms (*22*). Strategies and approaches to prevent and reduce intimate partner violence might occur across different levels of social-ecological interrelationships, such as engaging men and boys as allies (*22*,*62*); disrupting developmental pathways toward intimate partner violence; creating protective school, workplace, and neighborhood environments (21); teaching youths about safe and healthy relationships (*22*,*63*); empowering bystanders; and strengthening economic supports for families (*22*). Prevention efforts can help change harmful gender norms that condone violence and the societal conditions that serve to maintain those norms (*22*,*64*).

#### The COVID-19 Pandemic and Homicide

Research using NVDRS data to examine the circumstances of COVID-19 pandemic–related homicides are ongoing, but studies have already underscored the changes in homicide rates. The overall firearm homicide rate in 2020 was higher than it has been in the last 20 years (*1*,*45*). This increase was disproportionately experienced by AI/AN and Black persons (*1*). The increased social and economic stressors attributable to the COVID-19 pandemic and associated mitigation measures (e.g., job loss and disruptions in emergency services) might have exacerbated the systemic inequities that have been found to increase risk for experiencing violence (*45*,*54*,*55*). These stressors might also have increased risk for intimate partner violence and child abuse and neglect. Early pandemic reports indicated increased arrests and police calls related to intimate partner violence (*65*), and despite fewer visits, a higher proportion of emergency department visits were child abuse and neglect related (*66*). Additional research on the impact that the COVID-19 pandemic and mitigation measures might have had on increases in homicide rates and potential changes in the contextual factors and characteristics of these fatal incidents is needed.

#### Homicide Prevention Strategies

NVDRS programs have used their local VDRS data to examine homicides in their states to address state public health needs. For example, Illinois VDRS has used VDRS data to guide homicide prevention among youths in Chicago, where homicides are heavily concentrated among male and Black youths. Illinois VDRS published a study identifying contributing factors behind local fluctuations in youth homicides in the city of Chicago over a 10-year period from 2009 to 2018. The study examined associations between state funding of social and public health services, including welfare programs, housing subsidies, youth activity programming, mental health services, and other services, and risk factors for youth violence and homicides (*67*).

In conjunction with other data sources, NVDRS data can be used to can help states identify and address salient risk factors related to violence at the neighborhood and community levels, which can contribute to greater population-level decreases in violence through the reduction and elimination of systemic inequities (*58*). CDC’s A Comprehensive Technical Package for the Prevention of Youth Violence and Associated Risk Behaviors outlines multiple programs and approaches at the community and societal levels (*16*), such as street outreach programs (*68*), environmental design activities supporting safe spaces (*69*), business improvement districts (*70*,*71*), and policies that strengthen economic stability (*72*,*73*). For example, enhancing household financial security through tax credits such as the Earned Income Tax Credit can help families increase their income while also incentivizing work, counterbalancing the costs of child-rearing, and helping create home environments that encourage healthy development (*72*,*73*). Evaluations of these programs and policies have confirmed the value of using these types of approaches to reduce the risk for violence and promote protective community environments (*16*). Evidence also suggests that these approaches and other universal policies that focus on general community improvements can have a substantial effect on decreasing racial and ethnic inequities in violence (*21*).

The elevated homicide risk among AI/AN females has garnered national and political attention because of the underreporting of missing and murdered indigenous females in the United States (*74*–*76*). In 2016, the National Crime Information Center recorded 5,712 reports of missing AI/AN females, whereas the U.S. Department of Justice had only 116 such cases recorded in the same year (*74*). Two laws, Savanna’s Act and the Not Invisible Act, were enacted in 2020 to provide legal provisions to increase and improve data on the number of missing or murdered AI/AN persons, including AI/AN females (*77*,*78*). Approaches that improve data collection and access (e.g., improve racial classification of records, record-keeping, and sharing of records among and by law enforcement) and promote increased and accurate media coverage have been noted as meaningful ways to address violence against AI/AN females (*74*).

#### Legal Intervention Deaths

NVDRS collects more complete information on legal intervention deaths than other existing data sources (*79*). The rate of legal intervention death was highest among AI/AN persons, and the rate among Black males was 2.4 times that of their White male counterparts, a finding consistent with previous studies (*80*,*81*). Racial and ethnic inequities in fatal police shootings have been examined in the literature (*82*–*84*) and have been found to be associated with factors such as increased police contact because of more traffic stops, higher presence of law enforcement in racial and ethnic minority communities, and race-based bias and perceptions of threat. More analyses are needed to increase knowledge about the magnitude and circumstances of these deaths and for developing appropriate prevention strategies and monitoring their effectiveness. Multiple strategies have been proposed and reviewed to improve policing as possible ways of decreasing legal intervention deaths (*81*,*82*,*85*–*89*). For example, studies have suggested increasing training for law enforcement to reduce potential bias in interactions with suspects and training in conflict de-escalation and tactical disengagement as approaches to reducing legal intervention deaths (*81*,*82*).

In 2016, the U.S. Department of Justice Equal Opportunity Commission engaged in an initiative designed to help law enforcement agencies recruit, hire, retain, and promote officers who reflect the diversity of the communities they serve, all of which have been found to improve trust and relations between law enforcement and communities (*90*). NVDRS data have also been used at local levels to highlight racial and ethnic inequities in legal intervention deaths. In response to a Council of State and Territorial Epidemiologists priority to address law enforcement–involved fatal encounters and nonfatal injuries as a public health issue, New York VDRS used their data to examine legal intervention injuries and race and ethnicity (*91*). New York VDRS demonstrated that legal intervention deaths in New York from 2015 to 2017 largely affected Black persons (43%). Further, Black persons accounted for the majority of unarmed or bystander legal intervention deaths.

A unique strength of the NVDRS is the ability to collect data on characteristics of law enforcement officers involved in legal intervention deaths (*2*,*92*). Although not examined in this report, a previous study examining characteristics of officers involved in legal intervention deaths found associations between officer use of lethal force and characteristics such as race, age, sex, education, and previous use of force (*92*). Because of previous findings on characteristics of officers involved in legal intervention deaths and the importance of NVDRS for capturing information on legal intervention deaths, researchers have called on NVDRS to increase the completeness of demographic information on officers involved in these deaths (*81*,*92*).

#### Unintentional Firearm Deaths

NVDRS also has been recognized as a reliable source of data on unintentional firearm deaths (*93*,*94*) and for its ability to provide details about victims and shooters (*93*,*94*). In this report, approximately one half of unintentional firearm deaths were self-inflicted; however, approximately one third were inflicted by another person. Most of these deaths occurred while playing with a gun, unintentionally pulling the trigger of a gun, thinking a gun was unloaded, or mistaking a gun for a toy, which are concerning circumstances, particularly among children; these findings highlight the importance of safe storage practices and education about safe handling of firearms (*95*).

## Limitations

The findings in this report are subject to at least seven limitations. First, NVDRS data are available from a limited number of states, the District of Columbia, and Puerto Rico, and therefore are not nationally representative. In addition, California and Texas data were from a subset of counties and are not representative of all violent deaths occurring in these states.

Second, the availability, completeness, and timeliness of data depend on partnerships among VDRS programs and local health departments, vital statistics registrars’ offices, coroners and medical examiners, and law enforcement personnel. Data sharing and communication among partners are particularly challenging when states and U.S. territories have independent county coroner systems (rather than a centralized coroner or medical examiner system), numerous law enforcement jurisdictions, or both. NVDRS incident data might be limited or incomplete for areas in which these data-sharing relations are not fully developed. Partnerships with local vital statistics registrars’ offices usually are more established because they are part of the public health infrastructure. As part of an active surveillance system, VDRS programs work closely with local vital registrars’ offices to identify deaths that meet the NVDRS case definition and to avoid cases being missed or inappropriately included. CDC also monitors case ascertainment and variable completeness through regular technical assistance calls, which include reviews of the internal data quality dashboard in the web-based system that is updated in real time. Overall, core variables that represent demographic characteristics (e.g., age, sex, and race and ethnicity) and manner of death were known for >99% of cases.

Third, toxicology data are not collected consistently across all states, the District of Columbia, and Puerto Rico or for all alcohol and drug categories. In addition, toxicology testing is not conducted for all decedents; thus, percentages of decedents with positive results for specific substances might be affected by testing practices in coroner or medical examiner offices (*96*).

Fourth, abstractors are limited to the data included in the investigative reports they receive. In addition, reports might not fully reflect all information known about an incident, particularly for homicides and legal intervention deaths, when data are less readily available until a full investigation and adjudication are completed.

Fifth, case definitions present challenges when a single death is classified differently in different documents (e.g., unintentional firearm death in a law enforcement report, homicide in a coroner or medical examiner record, and undetermined on the death certificate). NVDRS abstractors reconcile these discrepancies using standard NVDRS case definitions and select a single manner of death based on all source documents (*7*).

Sixth, variations in coding occur depending on the abstractor’s level of experience. For this reason, CDC provides extensive abstractor guidance and training, a coding manual to promote standardized data collection (*7*), and data validation checks. As part of their internal data quality efforts, VDRS programs are required to reabstract at least 5% of cases to examine consistency in coding and identify training needs of data abstractors.

Finally, medical and mental health information (e.g., type of condition and whether the decedent was receiving treatment) often are not captured directly from medical records but from coroner or medical examiner records and the decedent’s family members and friends. Therefore, the completeness and accuracy of this information are limited to the knowledge of the informant.

## Future Directions

As a web-based surveillance system, NVDRS continues to evolve, with recent modifications incorporating additional functionality. For example, a bulk validation function has been added to the system that provides NVDRS states and jurisdictions the capability to generate error reports on demand, thus enabling data quality assessments more frequently than once a year. In addition, NVDRS is exploring the use of the County Health Rankings (CHRs) and the Social Vulnerability Index (SVI) to better understand and describe health inequities and their impact on violence. CHRs measure health outcomes and socioeconomic factors among other items (*97*). SVI examines socioeconomic status, household composition, race and ethnicity, language, housing, and access to transportation (*98*). Using these indicators in conjunction with NVDRS data might help identify racial and ethnic communities that are at disproportionate risk for violence and its effects. Finally, this report summarizes data on violent deaths that occurred in 2020 in 48 NVDRS states, the District of Columbia, and Puerto Rico. The goal is to include data for all 50 states in future reports.

## Conclusion

Public health surveillance is the foundation for public health practice (*99*). Monitoring the prevalence of violence-related fatal injuries, defining priorities, and informing violence prevention activities are essential parts of public health surveillance. In 2018, NVDRS received funding for nationwide expansion. Although not all VDRS programs’ data met the inclusion criteria to be included in this report, all 50 states, the District of Columbia, and Puerto Rico began participating and entering data in NVDRS starting in 2019, an important step toward achieving the goal of providing nationally representative data. This expansion makes violent death information available for local communities to develop prevention efforts and allow for the system’s capacity to measure the need for and effects of violence prevention policies, programs, and practices at the national level.

## Footnotes

* Frequencies and rates of violent deaths included in this report differ slightly from the frequencies and rates of violent deaths reported by CDC’s WISQARS, which excludes nonresident deaths that occur in participating states and the District of Columbia (occurrent deaths). NVDRS tracks both resident and occurrent violent deaths in the overall data set, and the numbers in this report reflect both. VDRS programs are expected to collect information on violent deaths among their residents, wherever they occur, and fatal violent injuries occurring within their borders irrespective of the decedent’s residence status. If the states of residence and injury occurrence are both participating NVDRS states, the state of injury occurrence is responsible for collecting the information. By making this differentiation of responsibility, duplicate reporting is avoided.

† The name of the Institute of Medicine was changed to the National Academy of Medicine, effective July 1, 2015.

§ To be included in NVDRS, deaths of undetermined intent must have evidence of the possibility that the intent was purposeful, including use of a weapon or other evidence that force was used to inflict the injury. The coroner or medical examiner is typically unsure whether the death was a suicide or unintentional.

¶ California began collecting data in 2005 but ended data collection in 2009. In 2020, 35 California counties (Amador, Butte, Colusa, Contra Costa, Fresno, Glenn, Humboldt, Imperial, Kern, Kings, Lassen, Lake, Los Angeles, Marin, Mendocino, Merced, Modoc, Mono, Orange, Placer, Sacramento, San Benito, San Diego, San Francisco, San Mateo, Santa Cruz, Shasta, Siskiyou, Solano, Sonoma, Stanislaus, Tehama, Trinity, Ventura, and Yolo) contributed data to NVDRS.

** In 2020, four Texas counties (Bexar, Dallas, Harris, and Tarrant) contributed data to NVDRS.

†† See e.g., 45 C.F.R. part 46, 21 C.F.R. part 56; 42 U.S.C. §241(d); 5 U.S.C. §552a; 44 U.S.C. §3501 et seq.

§§ Drug trade is defined as the buying, selling, or passing of drugs from one person to another in exchange for goods or money.
